# Cerebrospinal fluid proteome shows disrupted neuronal development in multiple sclerosis

**DOI:** 10.1038/s41598-021-82388-w

**Published:** 2021-02-18

**Authors:** Ellen F. Mosleth, Christian Alexander Vedeler, Kristian Hovde Liland, Anette McLeod, Gerd Haga Bringeland, Liesbeth Kroondijk, Frode Steingrimsen Berven, Artem Lysenko, Christopher J. Rawlings, Karim El-Hajj Eid, Jill Anette Opsahl, Bjørn Tore Gjertsen, Kjell-Morten Myhr, Sonia Gavasso

**Affiliations:** 1 Nofima AS, Norwegian Institute of Food, Fisheries and Aquaculture Research, Osloveien 1, 1430 Ås, Norway; 2 Rothamsted Research, Harpenden, Hertfordshire, AL5 2JQ UK; 3 Department of Clinical Medicine, University of Bergen, Bergen, Norway; 4 Neuro-SysMed, Department of Neurology, Haukeland University Hospital, Bergen, Norway; 5 Faculty of Science and Technology, Norwegian University of Life Sciences, 1430 Ås, Norway; 6 Center for Laboratory Medicine, Østfold Hospital Trust, Grålum, Norway; 7 Proteomics Unit (PROBE), Department of Biomedicine, University of Bergen, Bergen, Norway; 8 Laboratory for Medical Science Mathematics, RIKEN Center for Integrative Medical Sciences, Yokohama, Japan; 9 Center for Cancer Biomarkers (CCBIO), Department of Clinical Science, Precision Oncology Research Group, University of Bergen, Bergen, Norway; 10 Department of Medicine, Haematology Section, Haukeland University Hospital, Bergen, Norway

**Keywords:** Biochemistry, Biological techniques, Biotechnology, Cell biology, Computational biology and bioinformatics, Developmental biology, Immunology, Molecular biology, Neuroscience, Diseases, Health care, Medical research, Molecular medicine, Neurology, Pathogenesis

## Abstract

Despite intensive research, the aetiology of multiple sclerosis (MS) remains unknown. Cerebrospinal fluid proteomics has the potential to reveal mechanisms of MS pathogenesis, but analyses must account for disease heterogeneity. We previously reported explorative multivariate analysis by hierarchical clustering of proteomics data of MS patients and controls, which resulted in two groups of individuals. Grouping reflected increased levels of intrathecal inflammatory response proteins and decreased levels of proteins involved in neural development in one group relative to the other group. MS patients and controls were present in both groups. Here we reanalysed these data and we also reanalysed data from an independent cohort of patients diagnosed with clinically isolated syndrome (CIS), who have symptoms of MS without evidence of dissemination in space and/or time. Some, but not all, CIS patients had intrathecal inflammation. The analyses reported here identified a common protein signature of MS/CIS that was not linked to elevated intrathecal inflammation. The signature included low levels of complement proteins, semaphorin-7A, reelin, neural cell adhesion molecules, inter-alpha-trypsin inhibitor heavy chain H2, transforming growth factor beta 1, follistatin-related protein 1, malate dehydrogenase 1 cytoplasmic, plasma retinol-binding protein, biotinidase, and transferrin, all known to play roles in neural development. Low levels of these proteins suggest that MS/CIS patients suffer from abnormally low oxidative capacity that results in disrupted neural development from an early stage of the disease.

## Introduction

Multiple sclerosis (MS) is a serious disease of the central nervous system (CNS) characterised by accumulation of lesions with disrupted myelin and axonal damage in the brain and spinal cord^1–3^. Relapsing–remitting MS is characterized by lesions disseminated in both space (multiple locations in the CNS) and time (repeated episodes). Patients presenting with the first symptoms of MS without evidence of dissemination in space and/or time are diagnosed with clinically isolated syndrome (CIS)^4^. Miller and co-workers^4^ reported that between 30 and 70% of CIS patients are subsequently diagnosed with MS. Most relapsing–remitting MS patients (85–90%) convert over time to secondary progressive MS, which is characterized by steadily worsening disability. A primary progressive disease from onset with gradual accumulation of disability is seen in 10–15% of patients. This publication focuses on relapsing–remitting MS and CIS. For simplicity, we use the term MS and not relapsing–remitting MS in this publication.

MS is a heterogeneous disorder in terms of clinical features, genetics, pathogenesis, and response to therapies. The molecular basis of the disease is unknown, although various hypotheses have been suggested. The most widely accepted model is that MS is an autoimmune inflammatory disorder triggered in the periphery through immune dysregulation that causes demyelination^3^. However, other models consider inflammation as secondary to the initial pathological processes in the CNS. Tsunoda and co-workers suggested that viruses spread by axonal transport induce axonal injury in the CNS to trigger demyelination, inflammation, and lesion development^5^. Stys and co-workers hypothesized that MS is a disease initiated within the CNS by degeneration of the inner myelin sheath, which secondarily triggers inflammation^6–8^. They argue that models that consider inflammation as secondary are supported by several observations in MS brains including lesions with apoptotic oligodendrocytes without inflammatory cells^9^, abnormal lipid biochemistry in otherwise normal appearing brain tissues^10^, global alterations revealed by advanced spectroscopic methodologies before they become visible by commonly applied methods such as histochemical staining and conventional magnetic resonance imaging^11–13^, and epigenetic changes in pathology-free regions of multiple sclerosis–affected brains that influence oligodendrocyte susceptibility to damage^14^.

The cerebrospinal fluid (CSF) reflects immunological and other biological processes that take place within the CNS. The proteome patterns in the CSF therefore harbour extensive information on biological processes and pathological mechanisms of MS^15^. However, heterogeneity of the disease causes challenges. The aim of the present publication was to search for molecular signatures of MS within the CSF proteome pattern while considering the heterogeneity of the disease.

## Results

### Cohort 1

In the present study, we reanalysed our own published CSF proteomics study^16^ consisting of 779 CSF proteins from 37 MS patients and 64 controls. The controls were 50 individuals with other neurological disorders and 14 individuals with non-neurological diseases. Unless specifically stated, the controls included all 64 controls.

**Figure 1:**
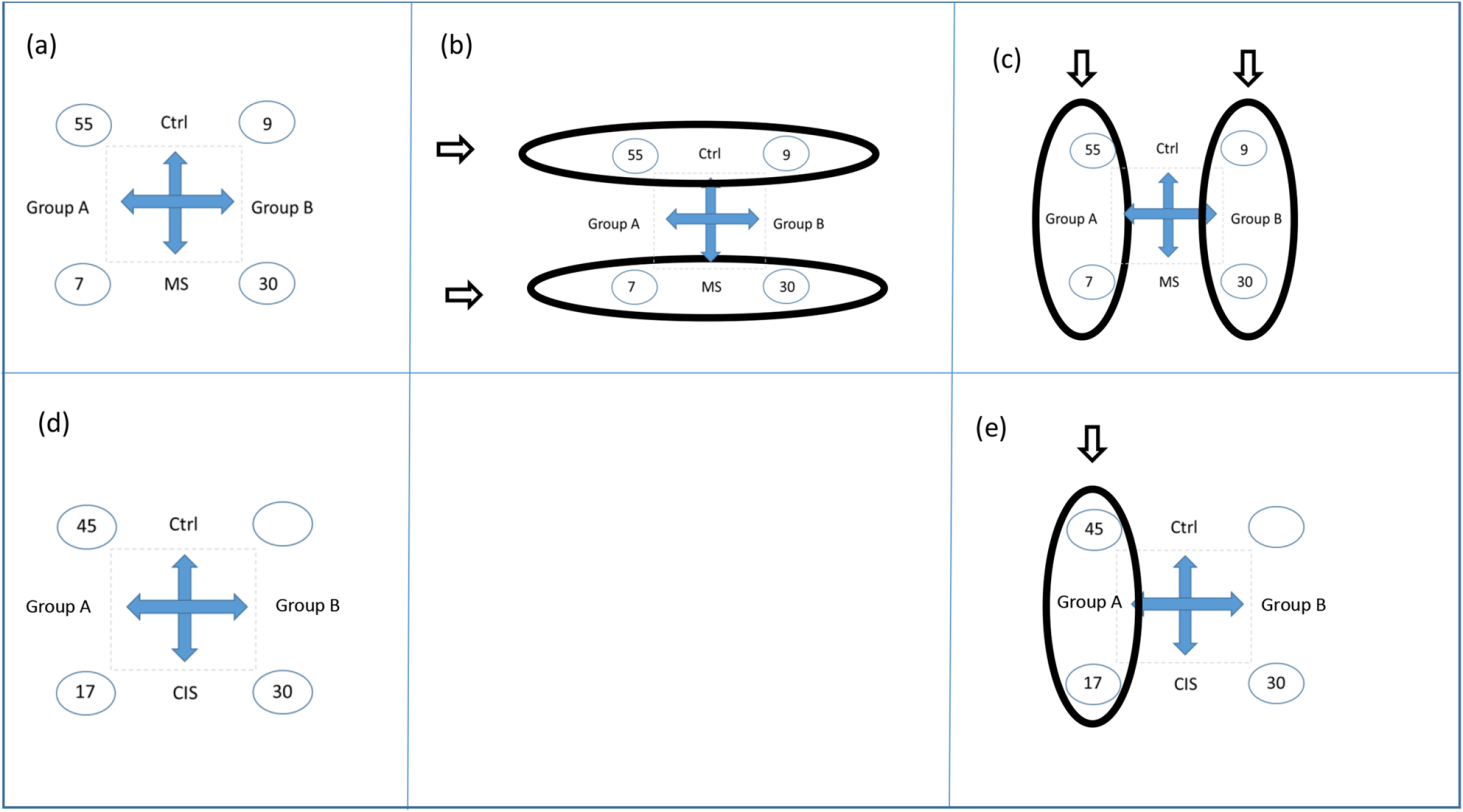
Cohorts 1 and 2. Design of the study. **(a–c)** Cohort 1: In our previous study of this material^16^ the 101 individuals in this cohort were separated based on proteome patterns into two groups here called groups A and B. (**a**) The data of this cohort is considered as influenced by two “pseudofactors”: Group A versus group B and MS versus control, resulting in four combinations of group affiliation and MS status. The four categories of individuals led to a two-factorial design for analyses. The numbers of individuals in each category are indicated in circles. (**b**) Confidence intervals of differences between the groups were analysed within MS patients and within controls. (**c**) Confidence intervals of the differences between MS patients and controls were performed within both groups. (**d** and **e**) Cohort 2: In analogy to cohort 1, two groups were established. Group A are the IgG-negative individuals. (**d**) For this cohort three combinations of group affiliation and CIS status were present. (**e**) Confidence intervals of the differences in the proteome patterns between CIS and controls were performed within group A.

In our previous analysis of cohort 1^16^, explorative multivariate analysis by hierarchical clustering separated the individuals into two groups with MS patients and controls present in both groups. The group with the most controls (55 controls and 7 MS patients) is in the present publication defined as group A, and the group with the most MS patients (30 MS patients and 9 controls) is defined as group B (Supplementary Table S1). Based on this, we consider two “pseudofactors”: the group affiliation (A or B) and the MS status (MS patient or control). Thus, four categories of individuals can be considered in a complete two-factorial design (Fig. 1a). The characteristics of the individuals in the four categories are given in Table 1a. It is important to take into account the differences in the number of individuals in each category, as the most frequent categories will dominate unless care is taken.

**Table 1:** Descriptive statistics of cohorts 1 and 2. Gender and the number of MS converters are given as numbers of individuals with percentage in brackets, and other values are given as means with standard deviations or ranges in brackets.

Cohort no	Sex total no	Group A		Group B	
		Controls	MS	Controls	MS
**(a) Cohort 1**					
Total number	101	55	7	9	30
Gender					
Females (%)	67	32 (58%)	6 (85%)	7 (78%)	22 (73%)
Males	34	23	1	2	8
Age (± SD) [years]		35 (9.9)	33 (4.7)	40 (9.2)	35 (6.9)
Protein concentration [µg/µL]		0.40 (0.11)	0.32 (0.07)	0.44 (0.18)	0.44 (0.10)
Time from first symptom to MS diagnosis [months]			Mean: 28 Range: 0–144		Mean: 24 Range: 0–192

		Controls	CIS	Controls	CIS
**(b) Cohort 2**					
Total number	62	45	17	0	30
Gender					
Females (%)	54	20 (44%)	11 (65%)		23 F (77%)
Males	38	25	6		7 M
Age (± SD) [years]		32 (9.4)	32 (11)		
Numbers of converters to MS (%)			5 (29%)		16 (53%)
Time from first symptom to MS diagnosis [months]			Mean: 29 Range 13–60		Mean: 32 Range 1–103

**Figure 2:**
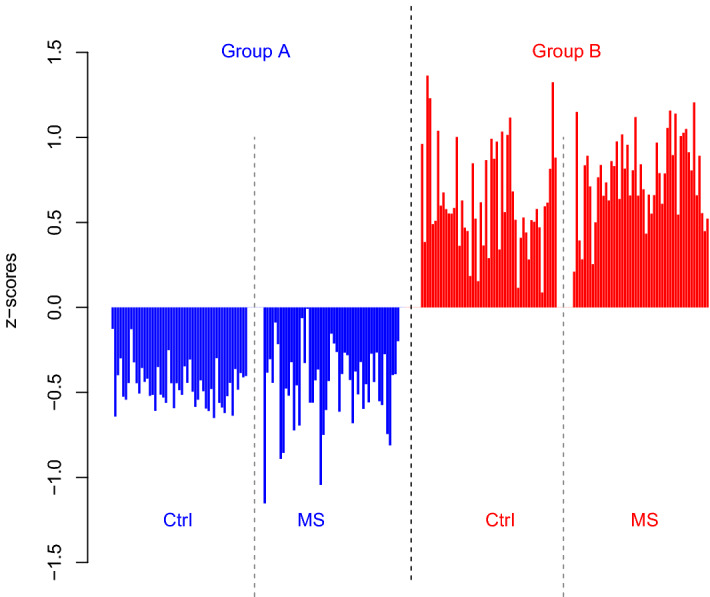
Cohort 1. Bar plots of IgGs significant by confidence intervals (95%) of the proteome for group A versus group B as analysed within MS patients and within controls. Each bar is the mean of one protein for one of the four categories: controls in group A (Group A, Ctrl, blue bars, n = 55), MS patients in group A (Group A, MS, blue bars, n = 7), controls in group B (Group B, Ctrl, red bars, n = 9), and MS patients in group B (Group B, MS red bars, n = 30). The identities of these IgG proteins are given in Supplementary Table S2. The y-axis is the abundance levels expressed as z-scores obtained by subtracting means and dividing by standard deviation.

In the present study we first analysed the differences between the groups. This analysis was performed by confidence intervals within MS patients and within controls (Fig. 1b), which revealed differential expression of 259 of the 779 proteins quantified (Supplementary Table S2) that were common in the analysis of MS patients and the analysis of controls. The majority of these proteins were increased in group B including most IgG proteins (Fig. 2). Thus, although most MS patients in both groups had positive IgG oligoclonal bands (Supplementary Table S1), MS patients in group A and B have significantly different patterns of IgG proteins in the proteome analysis. This led to the results of the hierarchic cluster analysis presented in our previous publication of this cohort^16^. Other proteins elevated in group B compared with group A were the fibrinogen proteins (FGA, FGB, and FGG). There were also some proteins detected at lower levels in group B than group A including cadherin EGF LAG seven-pass G-type receptor 2 (CELSR2), peroxiredoxin 2 (PRDX2), and immunoglobulin superfamily member 8 (IGSF8).

The aim of this study was to identify a common signature of MS within the two groups. To do this, we determined confidence intervals of the differences in the proteome patterns between MS and controls within both groups as illustrated in Fig. 1c. This identified a proteome signature of MS, unique to MS patients compared to controls and consistent within both groups (Supplementary Fig. S1, Supplementary Table S2). The same pattern was also identified in a separate analysis that included only the controls without neurological disorders (Supplementary Fig. S1).

For validation of the proteome analysis, two proteins, FGG and IGKC, were analysed by enzyme-linked immunosorbent assay (ELISA) in a subset of the patients (n = 24). The results showed the same pattern of variation as observed in the proteome analysis with correlation coefficients between the proteome analysis and ELISA of 0.90 for FGG and 0.85 for IGKC.

KEGG Pathway^17–19^ and Gene Ontology analyses of the proteins that were significant by the confidence analyses are shown in Supplementary Fig.S2. This graph is presented to reflect the consideration of the design as affected by two pseudofactors. The graph shows that proteins with increased levels in group B versus group A are involved in immune response, antigen binding, and peroxisome proliferator-activated receptor (PPAR) signalling pathways. Proteins with decreased levels in group B compared to group A are involved in purine and pyrimidine metabolism and G-protein coupled receptor signalling.

Proteins detected at decreased levels in MS patients versus controls within both groups were associated with the Gene Ontology terms CNS development, complement and coagulation cascades, response to wounding (i.e., bone morphogenetic protein signalling pathway), mineral absorption, extracellular matrix organisation, negative regulation of cellular iron ion homeostasis, insulin-like growth factor binding, and fat and vitamin digestion and absorption (Supplementary Fig. S2).

### Cohort 2

We also analysed CSF proteomics data from an independent cohort (cohort 2) obtained using a similar proteomics platform. This cohort consisted of 47 CIS patients and 45 controls^20^. Among the 47 CIS patients, 28 patients had positive oligoclonal IgG bands and/or increased IgG index > 0.67, 17 patients had normal CSF IgG levels, and two had unknown IgG status. Of the 47 CIS patients in this cohort, 21 were diagnosed with MS during follow-up. The mean time for conversion from CIS to MS was 31.4 months during a follow-up time of up to 117 months^20^. Among the 17 CIS patients with normal CSF IgG CSF levels, 5 converted to MS within the observation period (Supplementary Table S3).

For the analyses of cohort 2, we evaluated the 357 proteins that were quantified in both cohorts. Some proteins that were significantly lower expressed for MS versus controls within both groups in cohort 1 were not available in cohort 2, such as attractin (ATRN). Ig kappa chain C (IGKC) was the only protein in the proteome that was significantly increased in CIS patients compared with controls in the previous analysis of these data^20^. This protein was not increased among the 17 CIS patients with normal CSF IgG levels compared to controls (Supplementary Fig. S3). Thus, 17 CIS patients in cohort 2 had no evidence of elevated CSF IgG from the IgG index, from the oligoclonal IgG bands, or from the CSF proteome pattern. For cohort 2, we defined two groups of patients, A and B, analogous to the groups in cohort 1 (Fig. 1d). Group A patients are the 17 IgG-negative CIS patients and the 45 controls without any signature of intrathecal inflammation. Group B included the remaining patients and no controls. Characteristics of this cohort are presented in Table 1b.

In both cohorts, all CSF samples were taken prior to any disease modifying therapy. Furthermore, the length of the period from the first symptom to MS diagnosis did not differ significantly between the two groups in either cohorts. The two cohorts include patients at different stages of disease. Common to both cohorts is that there was no significant signature of ongoing intrathecal inflammation for patients in group A as validated by the proteome data.

Confidence interval analysis of CIS versus controls within group A of cohort 2 (consisting of 17 CIS patients and 45 controls, Fig. 1e, Table 1) were analysed for those proteins that were significantly decreased in MS patients relative to controls within both groups of as identified in cohort 1, and that were also present among the 357 protein that were quantified in cohort 2 (Supplementary Fig. S4). This analysis of cohort 2 revealed that the complement proteins significantly lower in abundance for MS versus controls in cohort 1 were also lower in CIS patients versus controls in cohort 2. Also expressed at lower levels in MS/CIS patients relative to controls were alpha-1B-glycoprotein, biotinidase (BTD), follistatin-related protein 1 (FSTL1), haptoglobin (HP), immunoglobulin superfamily containing leucine-rich repeat protein, inter-alpha-trypsin inhibitor heavy chain H2 (ITIH2), serum amyloid A-4 protein, and transferrin (TF). A separate analysis performed on the CIS patients in group A who converted to MS during follow-up compared to controls identified the same pattern of protein expression (Supplementary Fig. S4), although with a higher standard deviation due to the lower number of patients. In cohort 2, confidence interval could not be analysed within group B as this group consisted of only CIS patients. Decreased levels of apolipoprotein A-I (APOA1) and vitamin D-binding protein (GC) were significant for cohort 1 (Supplementary Fig. S1), but not for cohort 2 (Supplementary Fig. S4).

### Combining the data of all individuals within each group across cohort 1 and 2

The proteome data from the two cohorts were combined to perform confidence interval analyses within each group across the cohorts (Supplementary Fig. S5). This revealed a highly consistent pattern of variation as the analysis was performed by simply merging the data for the groups from the two independent cohorts. Thus, the proteins identified by analysis of the cohorts discriminated MS/CIS patients from controls irrespectively of intrathecal inflammation and irrespectively of the stage of the disease as CIS or MS. As there was more power in the statistical analysis of the combined cohorts due to the larger number of individuals, the number of significant proteins is higher for the combined data sets across the two cohorts than for similar analyses performed within the cohorts (Supplementary Figs. S1 and S4).

### Multivariate analysis within each cohort

**Figure 3:**
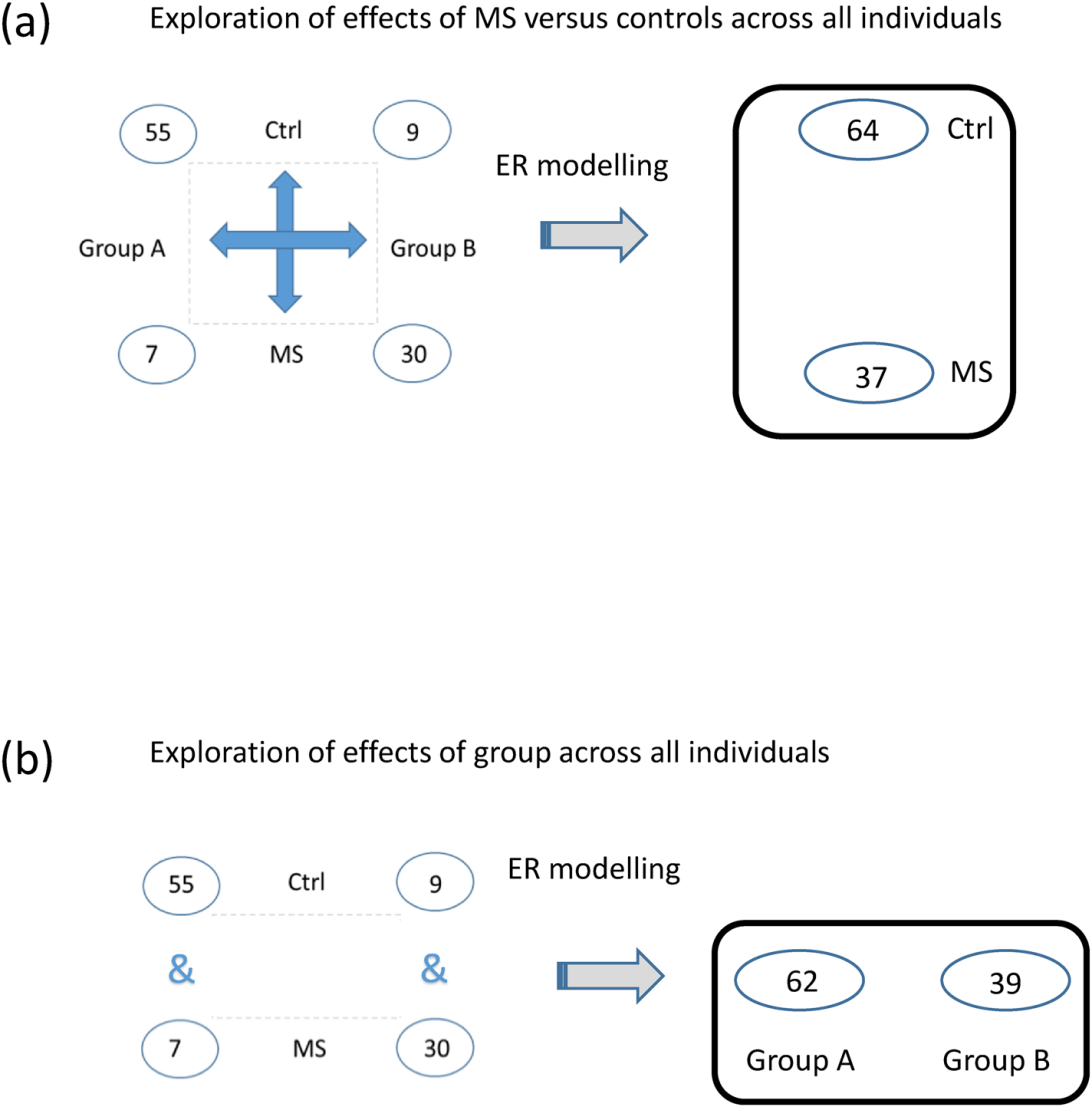
Cohort 1. Schematic description of ER modelling. The data are considered as two-pseudofactors, each on two levels, and the impact of one factor is omitted during exploration of the other. ER modelling allows data on all controls and all MS patients to be combined in multivariate analyses to identify (**a**) a disease-associated proteome pattern as well as (**b**) the proteome signature that drives group affiliation.

Next, multivariate analyses were performed to shed light on the underlying patterns of variation. As the data are influenced both by group and by MS status, a novel strategy was applied to isolate the effects of MS without confounding impact of group affiliation, and vice versa to analyse the effects of group without impact of MS status. Isolation of the effects was achieved by effect plus residual (ER) modelling^21^. The method is based on a linear model as in an ordinary two-way ANOVA, where the two pseudofactors, group affiliation and MS status, were used as the two input design factors. As in ordinary two-way ANOVA, this results in isolation of the effects of group affiliation and isolation of the effects of MS status for each protein. By ER modelling, we added the residuals of the model to each effect as illustrated in Supplementary Fig. S6. Applied on all proteins, this results in one data table of the proteome that reflects effects of group affiliation without influence of MS status, and one data table of the proteome that reflects effects of MS status without confounding impact of group affiliation. These ER values are provided in Supplementary Table S4. This approach allows multivariate exploration of group affiliation and multivariate exploration of MS status across the whole cohort (Fig. 3).

**Figure 4:**
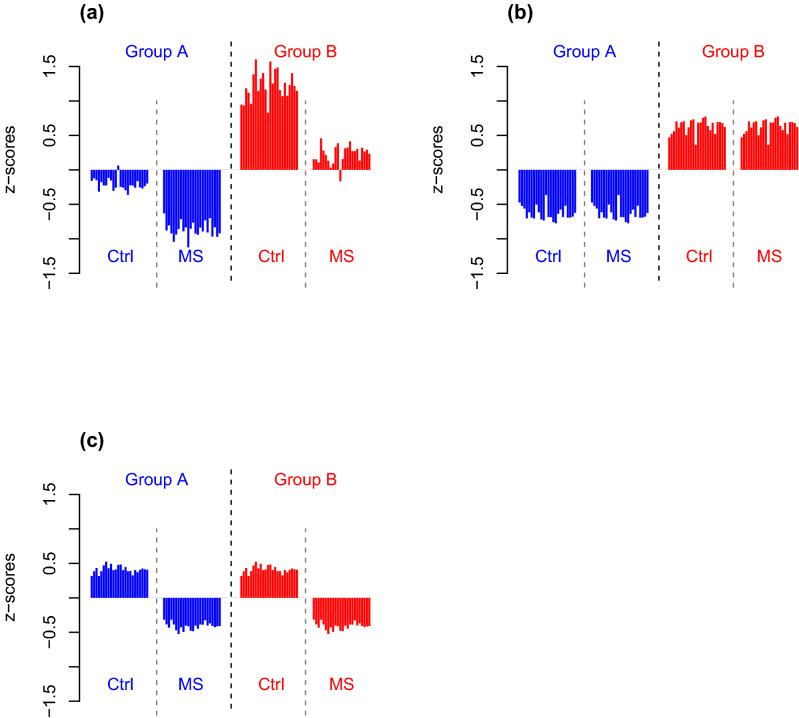
Cohort 1. Bar plots of proteins significant for MS versus controls by confidence intervals (95%) applied within group A and within group B. Each bar is means of one protein for each of the four categories: controls in group A (Group A, Ctrl, blue bars, n = 55), MS patients group A (Group A, MS, blue bars, n = 7), controls in group B (Group B, Ctrl, red bars, n = 9), and MS patients in group B (Group B, MS, red bars, n = 30). The protein identities are given in Supplementary Table S2. The y-axes are the abundance levels expressed as z-scores obtained by subtracting means and dividing by standard deviations. (**a**) Means of the data, (**b**) means of ER values of group affiliation obtained by ER modelling where impact of MS status is omitted to isolate the effects of group, and (**c**) means of ER values of MS status obtained by ER modelling where the impact of group affiliation is omitted to isolate effects of disease. The effects of group (panel **b**) plus the effects of MS status (panel **c**) gives predicted values (panel **a**). Importantly, the comparison of MS versus controls when group affiliation is ignored (as in panel **a**) is dominated by the most frequent categories, which are controls in group A (the blue bars to the left, n = 55) and MS patients in group B (the red bar to the right, n = 30), with the consequence that the lower abundance of these proteins for MS versus controls within group is not observed. Comparisons of MS versus controls based on ER values (displayed in panel **c**), which isolate the MS-specific effects, revealed the lower expression of these proteins for MS patients compared with controls without confounding impact of group affiliation.

The proteins that were decreased for MS versus controls as identified by confidence intervals within both groups had a dual pattern of variation (Fig. 4a). These proteins were detected at lower levels in MS patients than in controls in both groups, but they were also elevated in group B compared to those in group A. Thus, unless both group affiliation and MS status are considered, the group affiliation will cause confounding effects and mask the effects of MS. ER modelling solves this by isolating the effects of each factor. Described in another way, by ER modelling, the effects of group affiliation are omitted as an offset (Fig. 4b), which enables exploration of the effects of MS status without confounding impact of group affiliation (Fig. 4c).

Partial Least Squares-Discriminant Analysis (PLS-DA)^22^ was applied for multivariate analyses of ER values in cohort 1. PLS-DA, which belongs to the same family of methods as principal component analysis (PCA), projects the observed data onto underlying multivariate patterns, PLS factors, that are bi-linear functions of the original variables (i.e., protein quantities) where one or more response parameters are used to guide the projection. In our case, the response parameters are group affiliation or MS status. As in PCA, the PLS factors are described by coefficients (scores) of the individuals and corresponding coefficients of the proteins (loadings). The PLS factors reflect, in decreasing order, the variables relevant to the response parameter.

For cohort 1, multivariate analyses were performed on the ER values that isolated the effects of group affiliation using an indicator variable (–1 versus 1) of group A versus group B as response, and on ER values that isolated the effects of MS status using an indicator variable of MS versus controls as response. The proteins that were detected in both cohorts are included in this analysis. The multivariate analysis of cohort 2 only comprised data from group A, as group B did not have any controls. The data from group A of cohort 2 was analysed directly. Visual outputs of PLS-DA are displayed in Supplementary Fig. S7, and normality plots of residuals are displayed in Supplementary Fig. S8. The PLS-DA of group affiliation in cohort 1 separated the groups along the two first PLS factors, and the analysis of MS versus controls in cohort 1 resulted in separation of MS versus controls along the two first PLS factors as did the analysis of CIS versus controls in cohort 2.

PLS factors reflect the underlying multivariate pattern of variation relevant for the response. To guide the interpretation of this pattern, feature selection by Martens’ uncertainty test^23^ was performed. The signs of the regression coefficients in a model with two PLS factors were also considered in the search for a shared pattern across the two cohorts. This resulted in a selection of 44 proteins as a common signature of MS/CIS for the two cohorts, the majority with negative regression coefficients, indicating a protein pattern expressed at lower levels in MS/CIS than controls (Table 2, Supplementary Table S4). The proteins identified as significant for MS by confidence intervals within each group in cohort 1, and for CIS within group A in cohort 2 were among the proteins selected by Martens’ uncertainly test, with only one exception, HP, which was significant by univariate validation but not by the multivariate validation. Ontology analysis of the 44 proteins revealed enrichment in complement cascade, glucose metabolism, NAD and NADH metabolic processes, oxaloacetate metabolic process, generation of neurons, CNS development, bone morphogenetic protein signalling pathways, axon generation, axogenesis, axonal guidance, integrin-mediated signalling pathway, neuron cell adhesion, myelination, modulation of chemical synapsis transmission, positive regulation of long-term synaptic potentiation, regulation of transmitter receptor activity, and transition metal ion homeostasis (Supplementary Fig. S9).

**Table 2:** Proteins selected by Martens’ uncertainty test^23^ in the multivariate analysis PLS-DA for discrimination of MS/CIS versus controls with consistent negative regression coefficients in a model with 2 PLS factors.

Association Numbers	Gene names	Protein names	Regression coefficients^(a)^		*P* values^(b)^
			Cohort 1	Cohort 2	Cohorts 1 and 2
P04217	A1BG	Alpha-1B-glycoprotein	−0.015	−0.012	***
Q9P0K1	ADAM22	Disintegrin and metalloproteinase domain-containing protein 22	−0.012	−0.002	**
P05090	APOD	Apolipoprotein D	−0.007	−0.003	**
P02749	APOH	Beta-2-glycoprotein 1 (Apolipoprotein H)	−0.011	−0.004	***
P43251	BTD	Biotinidase	−0.015	−0.016	***
Q9NZP8	C1RL	Complement C1r subcomponent-like protein	−0.012	−0.011	***
P06681	C2	Complement C2	−0.012	−0.013	***
P01024	C3	Complement C3	−0.015	−0.006	***
P10643	C7	Complement C7	−0.008	−0.003	**
P00751	CFB	Complement factor B	−0.017	−0.010	***
P08603	CFH	Complement factor H	−0.007	−0.008	***
P05156	CFI	Complement factor I	−0.021	−0.008	***
P02452	COL1A1	Collagen alpha-1(I) chain	−0.005	−0.004	**
P08123	COL1A2	Collagen alpha-2(I) chain	−0.008	−0.002	**
Q16610	ECM1	Extracellular matrix protein 1	−0.021	−0.012	***
P02751	FN1	Anastellin	−0.021	−0.009	
Q12841	FSTL1	Follistatin-related protein 1	−0.013	−0.007	***
P22692	IGFBP4	Insulin-like growth factor-binding protein 4	−0.014	−0.004	***
P01880	IGHD	Ig delta chain C region	−0.024	−0.011	***
O14498	ISLR	Immunoglobulin superfamily containing leucine-rich repeat protein	−0.015	−0.009	***
P19823	ITIH2	Inter-alpha-trypsin inhibitor heavy chain H2	−0.011	−0.012	***
Q08380	LGALS3BP	Galectin-3-binding protein	−0.004	−0.003	**
P40925	MDH1	Malate dehydrogenase, cytoplasmic	−0.025	−0.007	***
P13591	NCAM1	Neural cell adhesion molecule 1	−0.011	−0.001	
O15394	NCAM2	Neural cell adhesion molecule 2	−0.021	−0.001	**
P19021	PAM	Peptidyl-alpha-hydroxyglycine alpha-amidating lyase	−0.020	−0.006	**
P30086	PEBP1	Hippocampal cholinergic neurostimulating peptide	−0.017	−0.005	**
P07225	PROS1	Vitamin K-dependent protein S	−0.003	−0.004	**
P41222	PTGDS	Prostaglandin-H2 D-isomerase	−0.002	−0.001	*
Q92932	PTPRN2	Receptor-type tyrosine-protein phosphatase N2	−0.009	−0.006	*
P02753	RBP4	Plasma retinol-binding protein (1–176)	−0.019	−0.006	***
P78509	RELN	Reelin	−0.012	−0.005	
P07998	RNASE1	Ribonuclease pancreatic	−0.011	−0.010	***
Q9BZR6	RTN4R	Reticulon-4 receptor	−0.002	−0.008	*
Q86UN3	RTN4RL2	Reticulon-4 receptor-like 2	−0.021	−0.005	*
P35542	SAA4	Serum amyloid A-4 protein	−0.014	−0.007	***
O75326	SEMA7A	Semaphorin-7A	−0.020	−0.011	***
P08294	SOD3	Extracellular superoxide dismutase [Cu–Zn]	−0.004	−0.005	*
P02787	TF	Transferrin	−0.020	−0.006	***
Q15582	TGFBI	Transforming growth factor-beta-induced protein ig h3	−0.006	−0.012	**

^(a)^Regression coefficients from PLS-DA within each cohort at two PLS factors.

^(b)^FDR-adjusted p-values (presented by one, two or three stars for *p* > 0.05, *p* < 0.01 and *p* < 0001, respectively) based on univariate validation by two-way ANOVA of the combined data of 163 individuals from cohort 1, groups A and B (after omitting the effects of group affiliation by ER modelling) and cohort 2, group A. The data on 357 proteins analysed in both two cohorts were considered.

#### Combined analysis of the two cohorts

**Figure 5:**
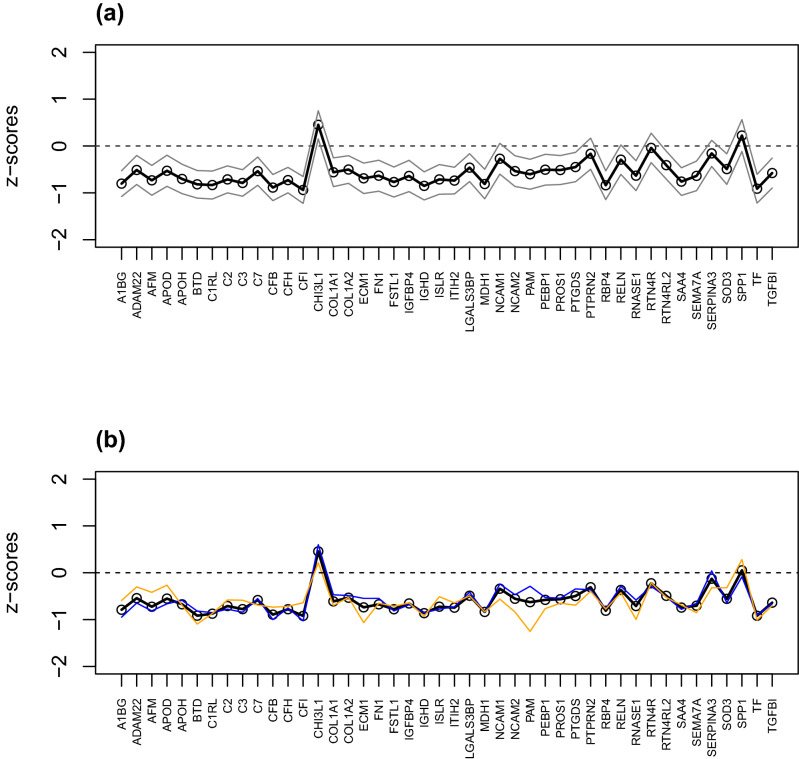
Cohort 1 and 2. (**a**) Confidence intervals (95%) of proteins selected by Martens’ uncertainly test in PLS-DA of the data from both cohorts. Confidence intervals are made on the merged data where the data table of ER values of MS status from cohort 1 are combined with the data of group A of cohort 2 into a single table of 163 individuals. The thick lines are the means, and the grey lines are the confidence borders. (**b**) Means of females (orange) and males (blue) and means of all (black). The protein names are provided in Supplementary Table S2 and S4.

The data table of ER values for MS status from cohort 1, which isolated the effects of MS versus controls, and the data of group A of cohort 2 were merged into a single table of 163 individuals (Supplementary Table S4). Calculations of confidence intervals of the differences between MS/CIS patients versus controls of the combined data showed that most of the identified proteins had decreased expression in MS/CIS compared to controls (Fig. 5). Further, this analysis demonstrated that differentially expressed proteins were similar in females and males (Fig. 5).

Two-way ANOVA was performed on the combined data of the 163 MS/CIS individuals by considering cohort affiliation (cohort 1 versus cohort 2), disease category (MS/CIS patients versus controls), and their interactions as inputs, and the quantities of the 357 common proteins as response. Of the 44 proteins selected by the multivariate analysis for MS/CIS versus controls within both cohorts, 37 were also significant by ANOVA considering p-values adjusted for false discovery rate (FDR) (Table 2, Supplementary Table S4). Thus, there was a consistency in the results from the multivariate analysis and the univariate analyses. This did not have to be the case as multivariate analyses consider the effects of the combination of proteins, whereas univariate analyses consider the effects of each protein one by one. Thus, in our dataset, most proteins selected by the multivariate analysis were also significant when considered one by one. When comparing the univariate analysis performed on the large data table of 163 individuals with the univariate confidence intervals performed within groups and within cohorts, the power of the statistics is larger with the larger number of individuals, which leads to more significant proteins. A more important consideration is that the confidence intervals performed within groups are designed to ensure that selected proteins are significant both for MS/CIS with intrathecal inflammation and for those without.

A multivariate approach enables consideration of combined impacts of all molecular fingerprints available. The family of multivariate analyses used in the present study has a more fundamental aspect. For multi-correlated features, as ‘omics data, PLS-DA projects information down onto an underlying pattern of variation, reflected by the PLS factors. It is interpretation of this underlying pattern of variation that can shed light on the biology that causes observed effects and gives rise to a correlated pattern of the observed features. In multi-correlated data, any feature selection procedure or statistical test is merely a guide in the search to understand this underlying pattern of variation.

A correlation plot of the selected proteins of the combined data of the two cohorts revealed strikingly close correlations in expression between most of the proteins selected by the multivariate approach (Supplementary Fig. S10). However, there were differences between patients and differences between the cohorts for some proteins. This is illustrated by considering the detailed patterns of expression of CFB, TF, and NCCAM2 (Supplementary Fig. S11). The three were all expressed at significantly lower levels for MS/CIS versus controls across the two cohorts. TF and CFB were closely correlated across the two cohorts, whereas NCAM2 displayed a different pattern that may reflect a different underlying regulatory mechanism. For some proteins, there were also differences between the cohorts. Examples are APOA1 and GC, which were significantly lower in abundance for MS patients versus controls in cohort 1, but not for CIS patients versus controls in cohort 2 (Supplementary Fig. S12). The intercorrelation between GC and APOA1 were strong within both cohorts, which suggests that these proteins have a common regulatory mechanism. Two other proteins, retinol-binding protein (RBP4) and BTD, were detected at significantly lower levels for the MS/CIS patients versus controls in both cohorts; however, levels of these proteins were not correlated with levels of APOA1 and GC, which may be due to a relationship to the stage of the disease or to individual differences. The low level of BTD for some of the CIS patients was not an isolated characteristic of BTD. The same CIS patients low for BTD were low also for other proteins that were identified as common characteristics for MS/CIS in the present study such as RBP4, TF, CFB, and NCAM2 (Supplementary Figs. S11 and S12).

### Multivariate analysis of MS status ignoring group affiliation

Data are normally analysed without considerations of the group affiliation such as that discovered in our previous study by explorative analysis of the proteome pattern^16^. To visualise how group affiliation can confound an observed pattern of variation, we performed multivariate analyses of cohort 1 without considering the grouping of individuals. A support vector model with feature selection^24^ was applied as described in Materials and Methods, and the results were visualised by PCA (Supplementary Fig. S13). This visualisation projects the main information onto principal components (PCs), which are bi-linear functions of the original data, with coefficients of the individuals (scores) and corresponding coefficients of the proteins (loadings). Using this method, the group affiliation dominated along the first and most important PC, even though the model was created to separate MS versus controls. Plots of individual proteins also revealed strong confounding impact of group affiliation (Supplementary Fig. S14). This analysis showed that when MS patients and controls were analysed without taking into consideration the group affiliation, the effects of group precluded identification of a disease-specific protein signature of MS.

## Discussion

**Figure 6:**
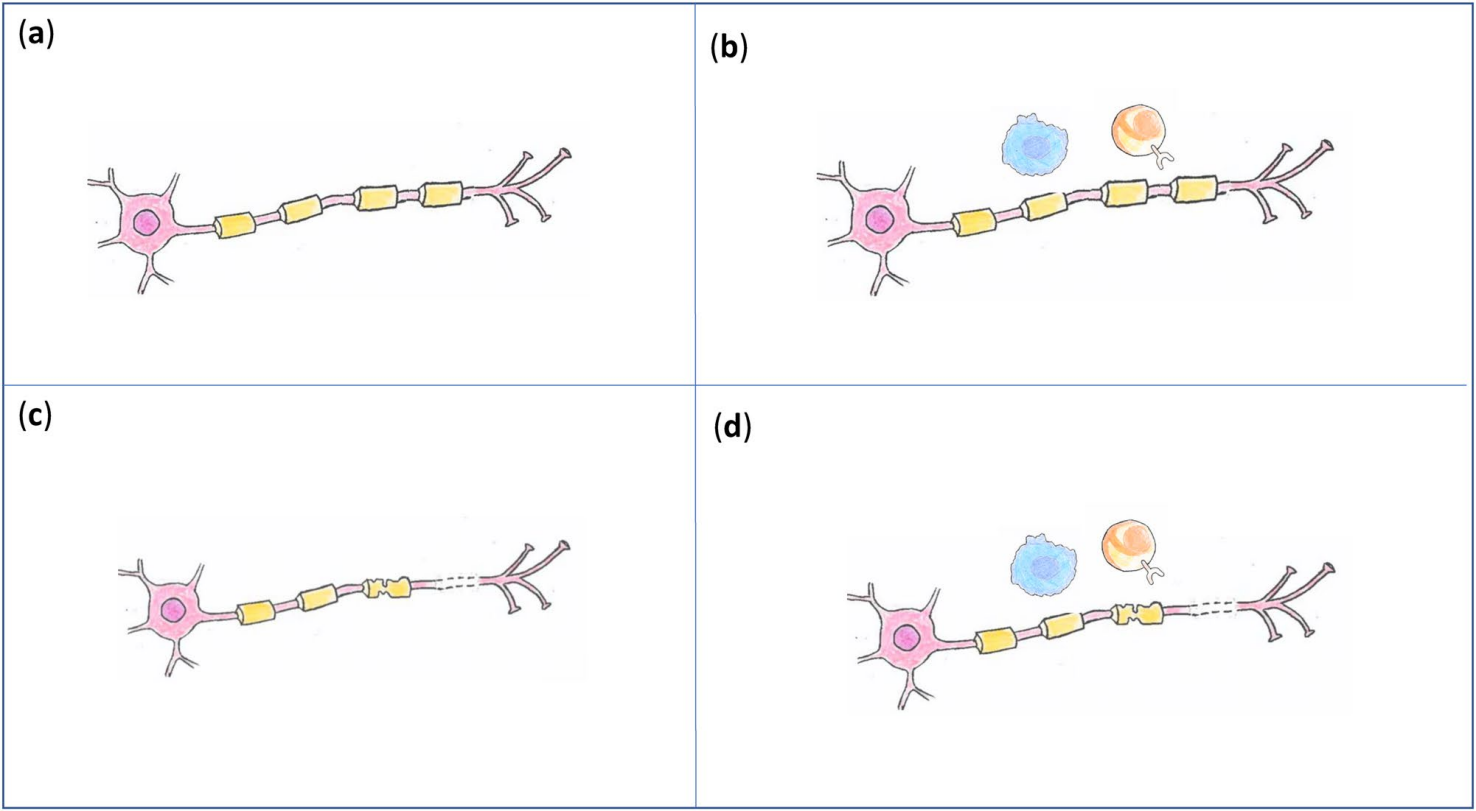
Four categories were considered based on disease status (MS versus controls) and group affiliation (group A and group B). The signature of intrathecal inflammation is simplified here by indicating only the immune cells. (**a**) Individuals without MS/CIS and without active intrathecal inflammation. (**b**) Individuals without MS/CIS with active elevated intrathecal inflammation. (**c**) MS/CIS patients without active intrathecal inflammation. (**d**) MS/CIS patients with active elevated intrathecal inflammation. Artwork performed by Kristiane Færgestad.

In this study we reanalysed previously published CSF proteome data from patients with MS (cohort 1)^16^ and CIS (cohort 2)^20^ and controls without these diagnoses. In previous work on cohort 1, explorative multivariate analysis of the CSF proteome separated the individuals into two groups with MS patients and controls present in both groups: One group but not the other had significantly increased levels of CSF IgG indicative of inflammation^16^. The strategy taken in the present study was to identify a common molecular signature of MS versus controls by removing the confounding influence of group. Cohort 2, which consisted of CIS patients and controls analysed with a similar proteomics platform^20^, was included in this study as an external data set. Among the CIS patients were patients with and without evidence of CSF inflammation, and, therefore, this cohort was also separated into two groups. The data represent four categories: controls in group A who do not have elevated intrathecal inflammation (Fig. 6a), controls in group B who have elevated intrathecal inflammation (Fig. 6b), MS/CIS patients in group A without elevated intrathecal inflammation (Fig. 6c), and MS/CIS in group B with elevated intrathecal inflammation (Fig. 6d). Categorisation allowed us to search for effect of MS/CIS that is consistent across the groups without confounding impact of group affiliation, which could otherwise mask the effects of MS/CIS. By our approach we identified a correlated pattern of the CSF proteins for MS/CIS patients that was consistent across the two cohorts and across the two groups.

The proteins that were decreased in abundance in MS/CIS patients relative to controls reflect an underlying mechanism of disturbed neural development present from the early phases of the disease. Among the proteins that were significantly decreased in CSF from MS/CIS patients with and without intrathecal IgG production compared with controls were the complement proteins C1RL, C2, CFB, CFI, C3, C7, and CFH. MS/CIS patients may have a predisposition to low complement levels or this may reflect chronic CNS infection^25,26^. Since Epstein Barr virus is a known risk factor for MS^27^, low levels of the complements may suggest that Epstein Barr virus infection could be involved in an early process that contributes to MS disease onset. Complement proteins are also involved in non-immune processes during CNS development, progenitor proliferation, neural migration, and synaptic pruning from the embryonic stage to the adult stage^28–32^. During synaptic pruning in the CNS, complement proteins tag redundant synapses for elimination, and research indicates that signalling mediated by transforming growth factor beta 1 (TGFB1) is involved in the process^29^.

TGFB1 was shown to be lower in abundance in MS/CIS compared with controls after omitting the confounding impact of group affiliation. TGFB1 is a pleiotropic signalling molecule^33^ with critical functions in neural development and homeostasis that function from the earliest stages of embryogenesis through adulthood^34,35^. TGFB1-mediating signalling is dependent on mitochondrial reactive oxygen species (ROS)^36,37^. Redox reactions, which involve the transfer or excitation of electrons in reversible oxidation–reduction reactions, are important for many cellular functions^38–47^. Within the CNS, redox signalling regulates physiological processes needed for neural development^38,41,47–63^, activation of neural progenitor cells under self-renewal^58,59,64^, maturation of neurons, signalling through extracellular matrix, and regulation of synaptic plasticity-related signalling molecules, receptors, and channels^38,65,66^. Importantly, redox balance toward oxidation is important in a healthy CNS^51,55–57,66^. Recently, Vicente-Gutierrez and co-workers showed in a transgenic mouse model that downregulation of endogenous mitochondrial ROS causes profound changes in brain energy and redox metabolism, leading to neural dysfunction and cognitive impairments^55^.

Two other closely correlated proteins that were decreased in MS/CIS versus controls were TF and RBP4. RBP4 binds retinol (vitamin A)^67^, an electron carrier in redox signalling^68^, known to play a central role in the control of energy homeostasis^69^, neural development^38,70^, and neural plasticity^71^. Upon demand for energy, retinol acts as catalyst in a reversible oxidation process to increase glucose-derived fuel flux into the citric acid cycle^69^, which implies a shift of the redox balance in the oxidative direction^51,68,69^. Whereas quiescent neural stem cells use glycolytic metabolism, neurons require more energy, and therefore a switch to aerobic mitochondrial respiration and oxidative phosphorylation is required during differentiation of neural stem cells^38,51,72^. The observed low level of RBP4 for MS/CIS may suggest disturbance in this process. TF binds and transport iron^73^. Iron can switch between the Fe^3+^ and Fe^2+^ oxidation states and is therefore an important co-factor for several redox enzymes including various enzymes critical for normal brain development and metabolism^38,52,74^.

BTD, another protein decreased in MS/CIS patients compared to controls, cleaves biotin (vitamin B7). Biotin is a B-complex vitamin essential for control of energy metabolism^75^ that promotes energy production and myelin synthesis in the CNS^76^. Biotin is a cofactor for several carboxylases in the citric acid cycle, which oxidizes biofuels to carbon dioxide and water, and thus is important for processes including fatty acid metabolism and carbohydrate metabolism^75^. Malate dehydrogenase 1 (MDH1), which was decreased in MS/CIS patients relative to controls, oxidizes the reduced form of nicotinamide adenine dinucleotide (NADH) to its oxidised form (NAD +) in the cytosol, making NAD^+^ available for the citric acid cycle in the mitochondria^77^.

The extracellular matrix provides structural support and also regulates many aspects of neural development through processes that involve redox signalling^78,79^. Follistatin-related protein FSTL1, which was also decreased in MS/CIS patients, is an extracellular glycoprotein that is involved in CNS development^80^. Depletion of FSTL1 in mice severely damages synaptic plasticity and causes altered expression of numerous genes involved in neurotransmitter transport, gamma-aminobutyric acid synaptic transmission, and synaptic plasticity^81^. ITIH2*,* which was also decreased in MS/CIS patients, contributes to extracellular matrix stability by covalent linkage to hyaluronan^82^. Hyaluronan acts both as a scaffold of the extracellular matrix and as a regulator of formation and function of synapse in developing neural networks^83^. Collagen alpha chain proteins COL1A1 and COL1A2 and extracellular matrix protein ECM1 were also decreased in MS/CIS patients versus controls.

Vitamin K-dependent protein ProS1^84^ was also decreased in MS/CIS. Vitamin K, which is expressed at high concentrations in brain cell membranes, participates in the synthesis of sphingolipids, which are components of cell membranes of oligodendrocytes and myelin. Sphingolipids and other membrane phospholipids serve as important structural components of membranes and function in cellular signal transduction, neuronal cell proliferation, differentiation, and synaptic transmission^85,86^. The effects of sphingolipids involve redox^87,88^, and there is a duality: ROS, reactive nitrogen species, and cellular redox potential are tightly linked to sphingolipid metabolism, and sphingolipids play important roles in maintaining cellular redox homeostasis^88^.

The neural cell adhesion molecules NCAM1 and NCAM2, which were also decreased in MS/CIS compared with controls, are cell-surface glycoproteins with large extracellular domains. The NCAMs mediate interactions between neurons and the extracellular environment by forming adhesive bonds with proteins located on neighbouring cells or by binding to proteins in the extracellular matrix. These proteins are normally abundant in the CNS and play roles in neural development, regulation of synapse formation, synaptic activity, and synaptic vesicle recycling at distinct developmental and activity stages^89,90^.

Disintegrin and metalloproteinase domain-containing protein ADAM22, a molecule that bridges the postsynaptic membrane and is known to play a key role in synapse maturation, synapsis transmission, and myelination^91^, was also decreased in MS/CIS patients compared to controls. As reviewed recently^92^, some ADAM family metalloproteinases process reelin, a large extracellular matrix protein that functions in the brain to regulate neuronal migration, dendritic growth and branching, dendritic spine formation, synaptogenesis, and synaptic plasticity^92,93^. Reelin was also down-regulated in MS/CIS patients.

Among other proteins that were decreased in MS/CIS patients compared with controls was semaphorin 7A. The semaphorins are signaling molecules^94^ that regulate the morphogenesis and homeostasis in a wide range of organ systems^95–97^. During neural development, semaphorins are involved in signaling necessary for axon guidance and neural morphogenesis, which are also redox-regulated processes^98–101^.

More proteins were quantified in the CSF of individuals in cohort 1 than cohort 2. Attractin ATRN is a protein that was detected as significant within both groups in cohort 1; it was not quantified in cohort 2. ATRN is known to have a critical role in normal myelination in the CNS. The myelination requires the coordinated synthesis of various structural proteins and enzymes, and ATRN serves as an anchor on the surface of neurons or glial cells where it mediates the myelination signal through its extracellular domains^102^.

The differences between the two groups of patients in cohort 1, which both include MS patients and controls, affected the expression levels of about one-third of the CSF proteins. Thus, there was a substantial shift in the proteome patterns of patients in the two groups of cohort 1, where MS patients and controls in group B, but not in group A, had increased levels of IgG and the fibrinogen proteins FGA, FGB, and FGG but decreased levels of proteins linked to compensatory mechanisms to reduce inflammation, such as proteins involved in regulation of the purine nucleotide catabolic process and the G-protein coupled receptor signalling pathway^103^. Increasing evidence highlights the central role of fibrinogens in promoting inflammatory processes within perivascular MS lesions; these proteins contribute to neuronal damage, and inhibiting tissue repair processes^104^.

The differences between MS patients in the two groups of cohort 1 were not related to the length of the period from the first symptom to the MS diagnosis. One hypothesis is that patients with different genetics and epigenetics, most likely related to the human leukocyte antigens^105–107^, respond differently to the same cellular signals that caused disrupted neural development and disturbed neural homeostasis. Thus, it may be that MS patients in group B responded with more inflammation than patients in group A to an underlying dysfunction. Another hypothesis is that the patients in the two groups are at different stages of the development of the disease. More data are needed to answer this question.

In the original analysis of the CIS data (cohort 2), axon-neuron proteins were shown to be expressed at decreased levels in the CSF of CIS patients compared with controls^20^. Our analysis of only the CIS patients with normal CSF IgG levels supports and extends these findings, as we discovered that proteins critical for normal CNS development are dysregulated in the CSF of CIS patients independently of intrathecal IgG synthesis, which corresponds to the results of cohort 1.

**Figure 7:**
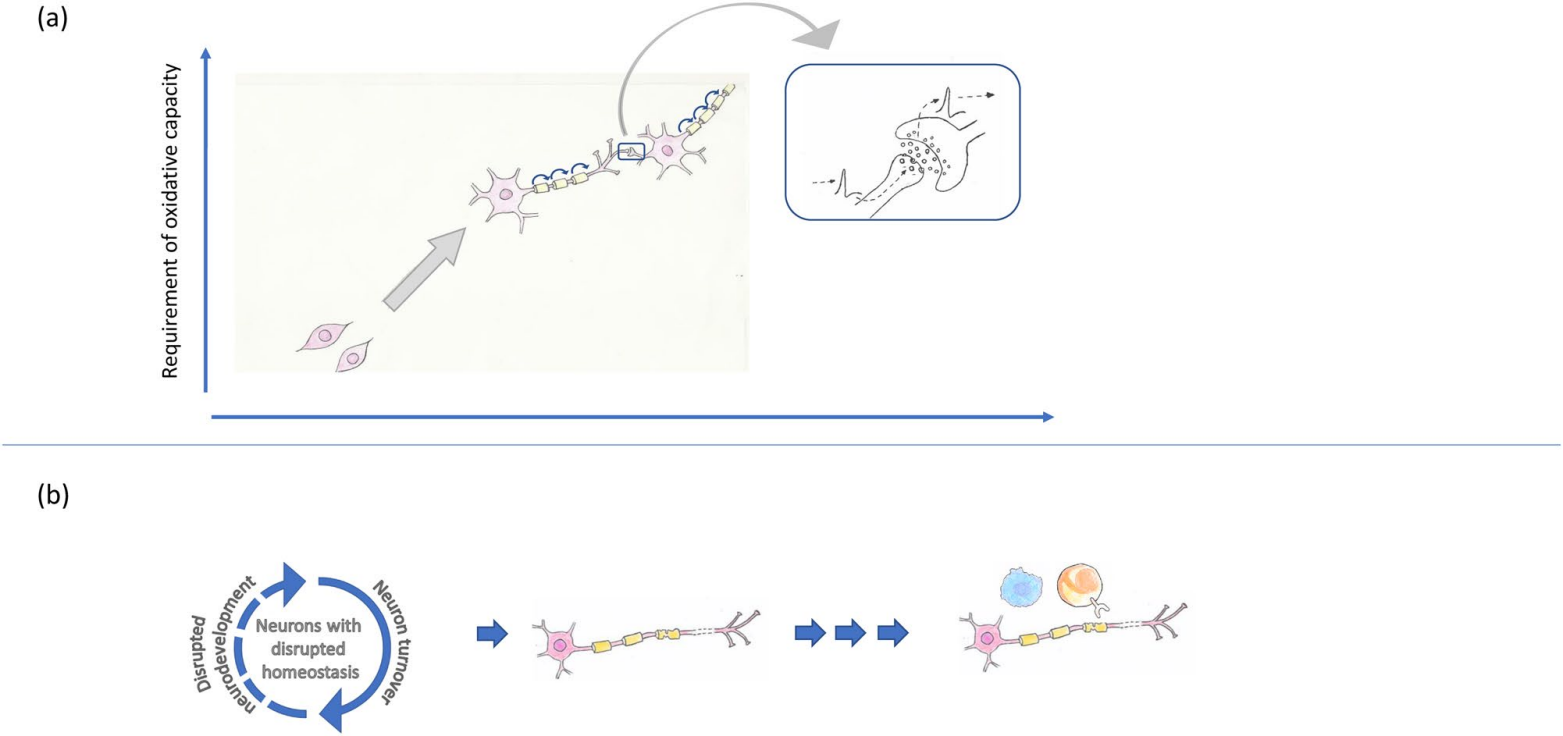
Normal neural development and function require oxidative capacity. (**a**) Requirement for oxidative capacity under normal neural development and homeostasis. (**b**) Loss of oxidative redox potential may be present from the early event in the pathogenesis of MS. We hypothesize that MS is a disease of disrupted neuronal development and homeostasis leading to the typical pathological characteristics of MS, and that inflammation is secondary. The signature of intrathecal inflammation is simplified here by indicating only the immune cells. Artwork performed by Kristiane Færgestad.

In summary, the proteome CSF pattern characteristic of MS/CIS that we identified supports the hypothesis that failure to generate sufficient oxidative redox potential is an important factor in neural health (Fig. 7). Loss of oxidative redox potential may represent an event present from the early stage of the pathogenesis of MS as this was observed also in CIS patients who have symptoms of MS without any evidence of intrathecal IgG synthesis. Thus, our analyses support the hypothesis of MS development presented by Tsunoda and co-workers^5^ and Stys and co-workers^6,108–110^ that CNS inflammation is a secondary event. However, their models suggest that MS is primarily a neurodegenerative disorder, whereas our results indicate that MS is a disorder of disrupted neural development. Considered in the context of adult neural development and neural homeostasis^111^, with dysregulated generation and turnover of myelin^112^ and neural proteins^113^, these findings lead us to hypothesize that disrupted neural development results in the typical pathological characteristics of MS.

**Figure 8:**
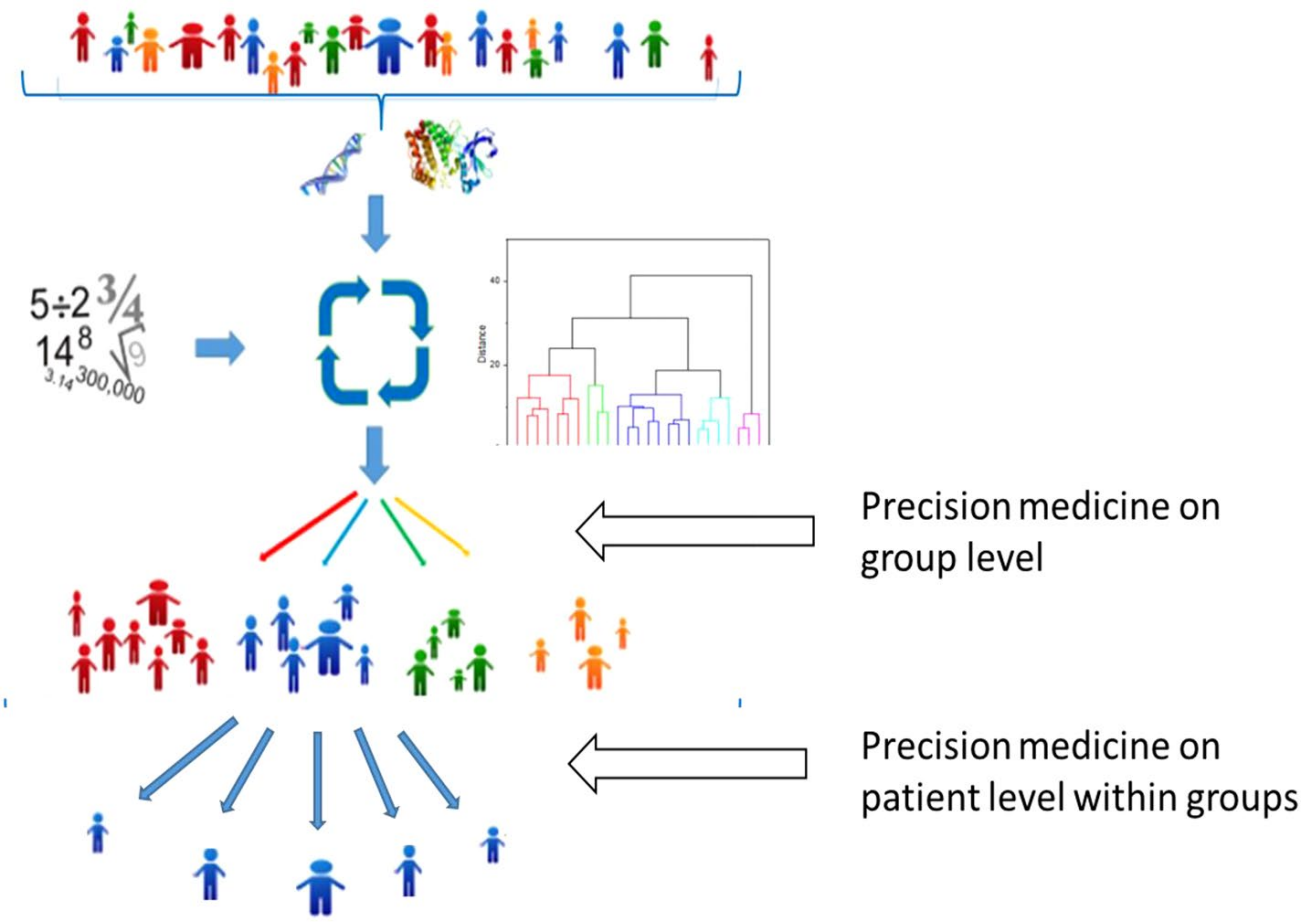
The ER modelling approach applied to precision medicine. In the present study we separated the participants into groups based on abundance of molecular markers and then analysed the data both within each group and across all participants using ER modelling to isolate the effects of group and to identify a disease-specific protein pattern. ER modelling can be utilized for precision medicine on group level to optimize therapy for patients in each group and to guide personalised medicine decisions within groups and likewise for other heterogenous data.

We suggest that future MS therapy should consist of a combination of agents: established immunomodulatory drugs and treatments to stimulate remyelination, as emphasised over the last decade^114^, and also stimulators of neurodevelopment in general. Furthermore, it will likely be important to focus on individual patient data. The new ER analytical method we applied in this study can be used to enable precision medicine on both group and individual levels, not only for MS but also for other heterogeneous diseases and data (Fig. 8).

In conclusion, results of our analysis of the CSF proteome suggest that failure in neural development, which disturbs normal neural homeostasis (development and repair), is a common event of MS present from the early stage. In contrast, secondary elevated inflammation occurs to various degrees among individual MS patients. Future studies should aim to identify strategies to compensate for this failure in neural development and repair.

## Materials and methods

### Materials

Two previously published cohorts of CFS proteomes were re-analysed in the present study. Cohort 1 consists of our previously published data on CSF proteomes obtained from patients with MS and controls^16^, where the study population consisted of 101 individuals, of whom 37 patients were diagnosed with MS and 64 were controls. Of the controls, 50 were diagnosed with other neurological disorders and 14 were neurologically healthy persons who had undergone spinal anaesthesia for orthopaedic surgery. Unless specified, all individuals without MS were considered as controls in the present study. Previously published^16^ stratification of the patients from an explorative multivariate analysis by hierarchical clustering was utilized in the present study: Group A had 7 MS patients and 55 controls, group B had 30 MS patients and 9 controls (Table 1). A single sample of CSF was analysed for each participant. The CSF samples were obtained from biobanks sampled at the following hospitals: i) Departments of Neurology and Anaesthesia & Surgical Services, Haukeland University Hospital, Bergen, Norway; (ii) Neurology Department UCL, Universite Catholique de Louvain, Brussels, ´ Belgium; (iii) Laboratory of Neuroimmunology, IRCCS, “C. Mondino” National Neurological Institute, Pavia, Italy; and (iv) Department of Anaesthesia and Surgical Services, Haukeland University Hospital, Bergen, Norway. All hospitals are members of the BioMSeu network for biomarkers in MS (http://www.biomseu.com). Samples from all available individuals were included in the proteome study without any data exclusion.

The proteome data of cohort 1 was first pre-processed as described in our original study by normalisation on the median intensities of a set of proteins considered as CNS-specific proteins^16^. In the present study, we thereafter scaled the data to means of zeros and standard deviations of one to give z-scores.

Cohort 2 is previously published data from Stoop and co-workers^20^. This cohort was obtained from the Erasmus MC University Hospital where all patients 18 to 50 years-of-age presenting with a first episode suggestive of demyelination were followed prospectively; informed consent was given by all patients. The cohort included 47 CIS patients and 45 controls. CSF samples, MRI, and clinical data were collected within 2 months after first symptom onset. Samples from all available individuals were included in this proteome study without any data exclusion. Clinically definitive diagnosis of MS was made if there was clinical evidence of dissemination in space and time. In the present study, we determined means over multiple peptides without missing values reflecting the same protein. We analysed all proteins that were also available in cohort 1. In the present publication, we scaled the data to means of zero and standard deviation of one to give z-scores.

Our analysis of cohort 1 revealed that group stratification in cohort 1 reflected differences in inflammatory proteins, which were significantly elevated in group B but not in group A. We defined two groups in cohort 2 based on IgG bands and IgG index. The presence of many IgG-negative CIS patients in cohort 2 allowed us to investigate the effects of CIS versus controls among patients with early signature of the disease without evidence of elevated CSF inflammation.

### Method details

#### Proteome analyses

Details of the proteome analyses are described in the respective original publications of cohort 1^16^ and cohort 2^20^.

#### ELISA of two selected proteins

FGG and IGKC were analysed by ELISA using commercially available kits (Kappa Human ELISA kit from Abcam ab157709, lot: GR3174712-5; Fibrinogen Human ELISA kit from Abcam, ab108841, lot: GR3177851-6) in a subset of the individuals to confirm the pattern of variation observed in the proteome analysis. The analyses were performed as described in the protocol.

#### Quantification and statistical analysis

Both univariate and multivariate selection criteria were used to guide the unravelling of the underlying phenomenon of the data. Overview over design and analysis performed on cohort 1 and cohort 2 is displayed in Table 1, Fig. 1 and 3.

#### ER modelling

ER modelling builds on known methods that combine linear models as in ANOVA with multivariate analysis^21,115–117^. Here we applied ER modelling to heterogeneous data with two ‘pseudo-factors’ as main effects: group affiliation and MS versus controls. The interaction term was omitted as it did not reveal significant impact. ER modelling is described in Supplementary Fig. S15, with R codes provided in Supplementary Fig. S16. Equation 4 in Supplementary Fig. S15 is visualised for cohort 1 in Fig. 4 for the proteins significant by confidence intervals of one factor within both levels of the other factor.

In ER modelling, linear models are applied as in a two-way ANOVA model, for each protein with group affiliation and the disease category (MS versus controls) considered as two “pseudo-factors”. As in ANOVA, a linear model estimates the effects of each factor and the residuals of the model. In ER modelling, the residuals of the complete model are added to the effects of each factor, hence the name ‘effects plus residual (ER) modelling’. The ER modelling method isolates one factor at a time while utilizing the residuals of the complete model to output two new data tables. In our case, one table reflected isolated effects of group affiliation and one reflected isolated effects of MS, both with the residuals of the complete model available for validation. ER modelling is implemented using an R program available on CRAN (https://cran.r-project.org/web/packages/ER/index.html).

#### Univariate analysis of the proteome considering two pseudo factors: group affiliation and MS

Univariate analyses were applied by confidence intervals (95%) on one factor at a time (group and MS) within both levels of the other factor. Thus, confidence intervals were performed on the difference between group B versus group A both within MS patients and within controls, and on the difference between MS versus controls within each group. These analyses were performed in the R program for ER modelling as described in Supplementary Fig. S15, although the same analysis could have been applied by confidence intervals of each factor within both levels of the other factors. Proteins found to be significant for MS versus controls in cohort 1 were validated by confidence intervals in cohort 2 as external cohort. The two cohorts were also combined within each group, simply by merging the data table, and confidence intervals of the differences between MS/CIS and controls were analysed within group across the two cohorts.

The data was also combined across the two groups where data of cohort 1 was included as ER values of MS status after omitting the impact of group affiliation, and only group A was included from cohort 2, resulting in a large data table of 163 individuals. Univariate analysis was performed as two-way ANOVA on this data of 163 individuals with cohort affiliation and disease category (MS/CIS versus controls) as two input factors. The p-values were FDR-adjusted using rotation methodology^118^. This method was chosen as this test allows evaluation of multicollinear data under the assumption of normality. The program FFMANOVA, written in R and available on CRAN (https://cran.r-project.org/web/packages/ffmanova/index.html), was used to adjust p-values by rotation test^119^. The univariate analyses were programmed in R (version 4.0.0) and RStudio (version 1.2.5019).

#### Multivariate analysis of the proteome considering two factors: group affiliation and MS status

To identify relevant features in cohort 1 in a multivariate context we applied PLS-DA^22^, which analyses the multivariate pattern related to each factor, group affiliation and MS or control, one factor at a time to avoid the confounding impact of one factor on the effects of the other. For multivariate feature selection, we applied Martens’ uncertainly test^23^, which is a modification of the original jackknife procedure developed for full-rank multivariate models^120^ adapted by Martens and Martens to bilinear models. The method performs a t-test of the regression coefficients across the cross-validation samples, which results in a selection of features based on the stability of the regression coefficient when one segment at the time is omitted from the calibration data and used for validation. In our analysis we used sample from one individual at the time in the cross-validation segments. When performing this test in ER modelling, using the ER package in R, the degrees of freedom is adjusted for the terms included in the linear model. PLS-DA was performed on the 357 proteins identified in both cohorts. The analysis of cohort 1 was applied after ER modelling, whereas the cohort 2 was analysed directly. The ER analysis and PLS-DA were programmed in R (version 4.0.0) and RStudio (version 1.2.5019).

#### Analysis of MS status ignoring the group affiliation

In cohort 1, multivariate analyses of all 779 available proteins were applied for the comparisons of MS versus controls ignoring the group affiliation. Recursive feature elimination with cross validation was validated exploratively using three different models^121^ to search for optimal discrimination with minimum number of proteins: (1) a logistic regression model with ‘Limited-memory Broyden, Fletcher, Goldfarb, Shanno algorithm’ (LBFGS) as solver, (2) a logistic regression model with Library for Large Linear Classification (LIBLINEAR) as solver, and (3) a support vector model (SVC) classifier with standard scikit-learn solver^24^. The analyses were performed on 90% of the patients as training data with the remaining 10% viewed as test data, randomly selected while maintaining same class proportions. This analysis is included in the publication for demonstration of the confounding effects of group affiliation when searching for MS specific protein pattern. The optimal model for discrimination of MS versus controls was obtained by SVC (model 3). PCA was performed to visualise the multivariate pattern of variation of selected proteins^21,116^. These analyses were performed in Python version 3.6.

#### Protein identification

Protein and gene names are from the original study of Opsahl and co-workers^16^ and were assigned using the R program “gProfiler2” available on https://cran.r-project.org/web/packages/gprofiler2/index.html.

Two enrichment analyses^122,123^ are presented, one made of the proteins validated in cohort 1 as significant by confidence intervals for group affiliation within both disease status (MS and controls), and for MS status within both groups. The other enrichment analysis is presented for proteins identified by multivariate analysis of MS/CIS versus controls performed within each cohort. The former enrichment analysis present Gene Ontology information on biological process, molecular process and KEGG pathway analysis using CytoScape ver. 3 from the UniProtKB database and metabolic pathway membership data from KEGG database. Graph annotations were performed using the web-service interfaces of these databases, which were accessed using UniProt.ws and KEGG.db R packages from Bioconductor (ver. 3.6), respectively. The second enrichment Gene Ontology information on biological processes is obtained using Enrichr (http://amp.pharm.mssm.edu/Enrichr/)^123,124^, and results were visualised using CytoScape ver. 3.

## Supplementary Information

Figure S1.

Figure S2.

Figure S3.

Figure S4.

Figure S5.

Figure S6.

Figure S7.

Figure S8.

Figure S9.

Figure S10.

Figure S11.

Figure S12.

Figure S13.

Figure S14.

Figure S15.

Figure S16.

Table S1.

Table S2.

Table S3.

Table S4.

Figure S17.

Supplementary Legends.

## Data Availability

**Materials availability** The present study involves reanalyses of previous publications^16,20^.

## Data and code availability

Cohort 1 proteome data were previously published by Opsahl and co-workers^16^, and cohort 2 data were previously published by Stoop and co-workers^20^, downloaded from Supplementary Material “prca1891-sup-0002-suppmat2” where we include all proteins available also in cohort 1. The data from the two cohorts are available as Excel files (DM.1, DM.1.c, and DM.2.c) and as R files. The Excel sheets present the ‘pseudofactors’ group affiliation (factor 1) and disease status, MS versus controls (factor 2) and the proteome data after mean centring and scaling to z-scores. The file DM.1 is the proteome data in cohort 1 for all available 779 proteins, DM.1c is the proteome data in cohort 1 for the proteins that are common proteins for the two cohorts, DM.2c is the proteome data in cohort 2 for the common proteins of the two cohorts. https://nofima.no/filearchive/publications/2020/multiple_sclerosis/DM.1.xlsxhttps://nofima.no/filearchive/publications/2020/multiple_sclerosis/DM.1.c.xlsxhttps://nofima.no/filearchive/publications/2020/multiple_sclerosis/DM.2.c.xlsx. The R files are available as arrays that can be plugged directly into the R codes described in Supplementary Fig. S16. The file my.array.1.RData is the proteome data for cohort 1 for all available 779 proteins, my.array.1c.RData is the proteome data for cohort 1 for the common proteins of the two cohorts, my.array.2c.RData is the proteome data for cohort 2 for the common proteins of the two cohorts. https://nofima.no/filearchive/publications/2020/multiple_sclerosis/my.array.1.RData. https://nofima.no/filearchive/publications/2020/multiple_sclerosis/my.array.1.c.RData. https://nofima.no/filearchive/publications/2020/multiple_sclerosis/my.array.2.c.RData

## Code availability

Program code for ER modelling, is available on CRAN (https://cran.r-project.org/web/packages/ER/index.html), and running scripts are given in Supplementary Figs. S15 and S16. Examples of the python code used for the multivariate analysis of MS status ignoring group affiliation are shown in Supplementary Fig. S17. The code can be run from Jupyter Notebook. The code is also available on Google Colab via this link:https://colab.research.google.com/drive/1Vqvb-Ie3u6qihwSGLT0u3A0ROqShNLeU?usp=sharing. The code can be run from the link on Google Colab. The user will be asked to sign in to a google account.
