# Supplementary figures and images for: Cerebrospinal fluid proteome shows disrupted neuronal development in multiple sclerosis
Source: Sci Rep. 2021 Feb 18;11:4087. doi: 10.1038/s41598-021-82388-w (PMC7892850; doi:10.1038/s41598-021-82388-w)

**(a)**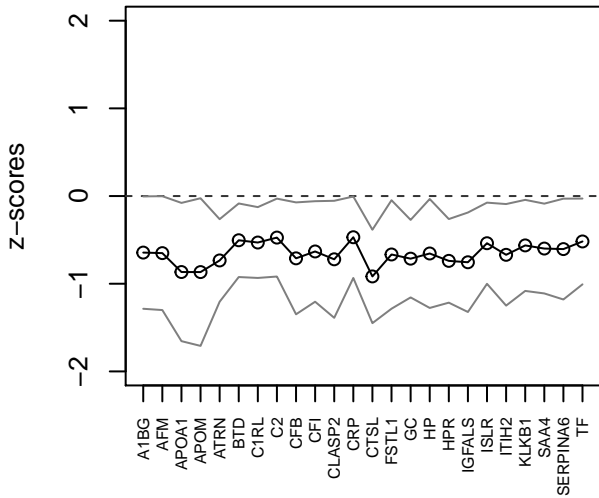**(b)**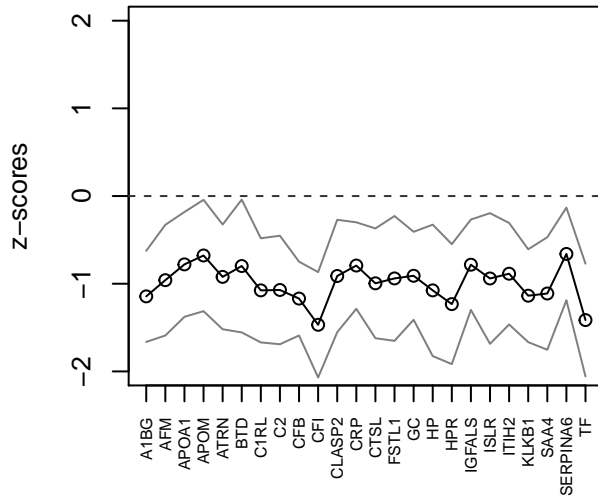**(c)**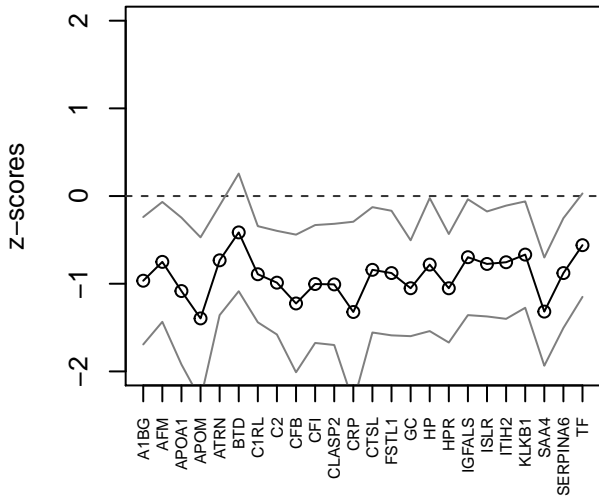

Supplement: Supplementary file 1 — Figure S1. [file 41598_2021_82388_MOESM1_ESM.pdf]

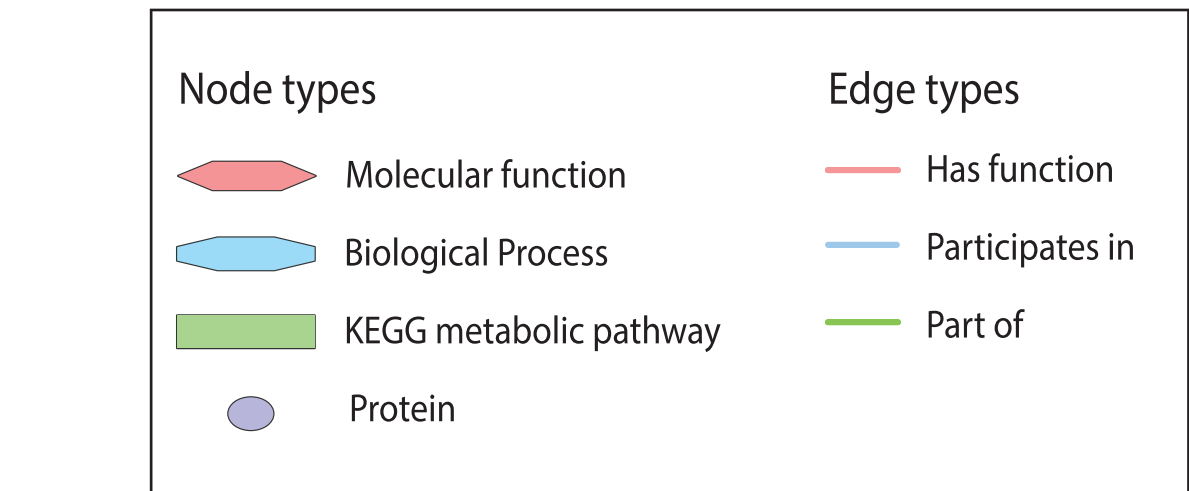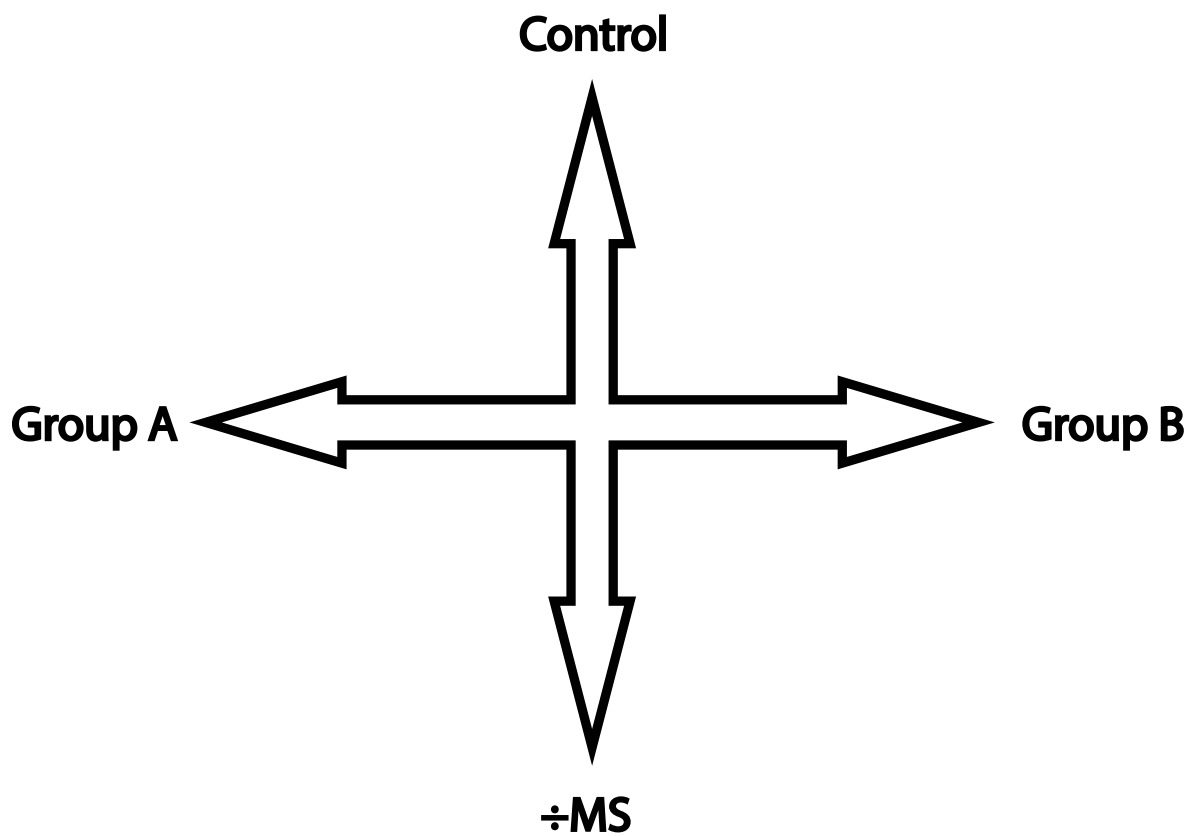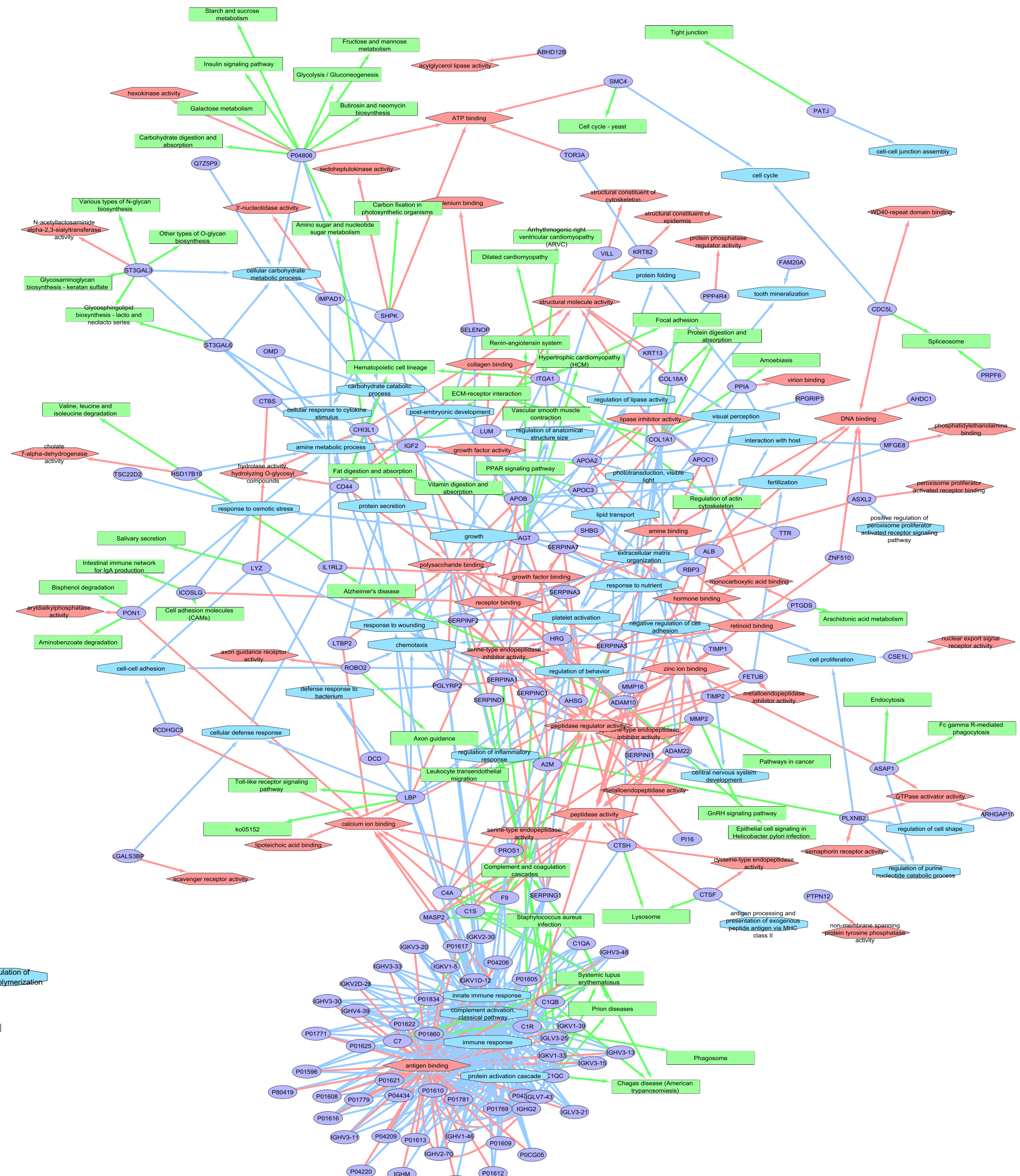

Supplement: Supplementary file 2 — Figure S2. [file 41598_2021_82388_MOESM2_ESM.pdf]

**(a)**

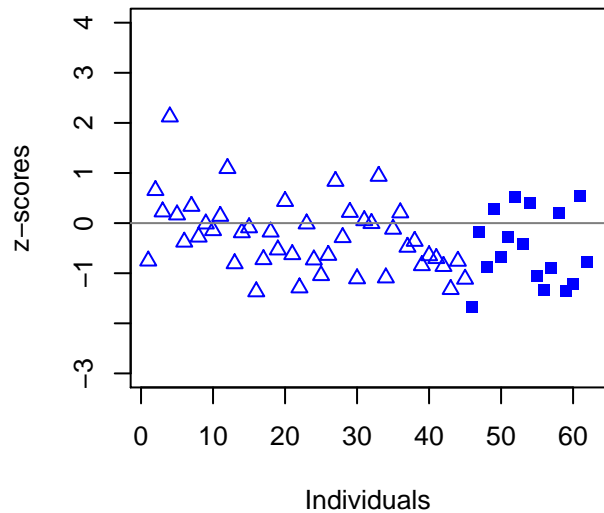

**(b)**

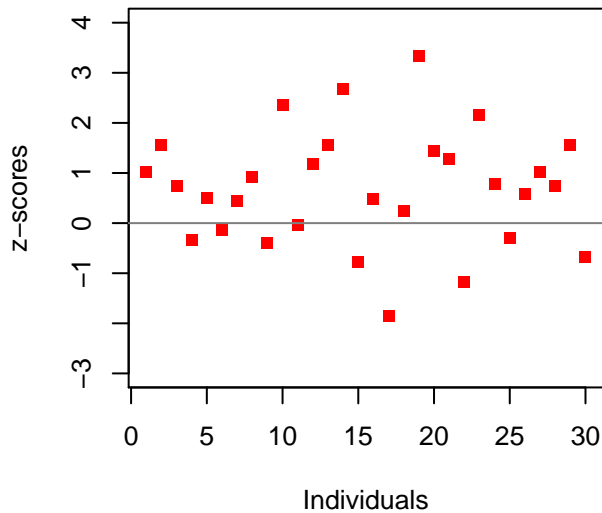

Supplement: Supplementary file 3 — Figure S3. [file 41598_2021_82388_MOESM3_ESM.pdf]

**(a)**

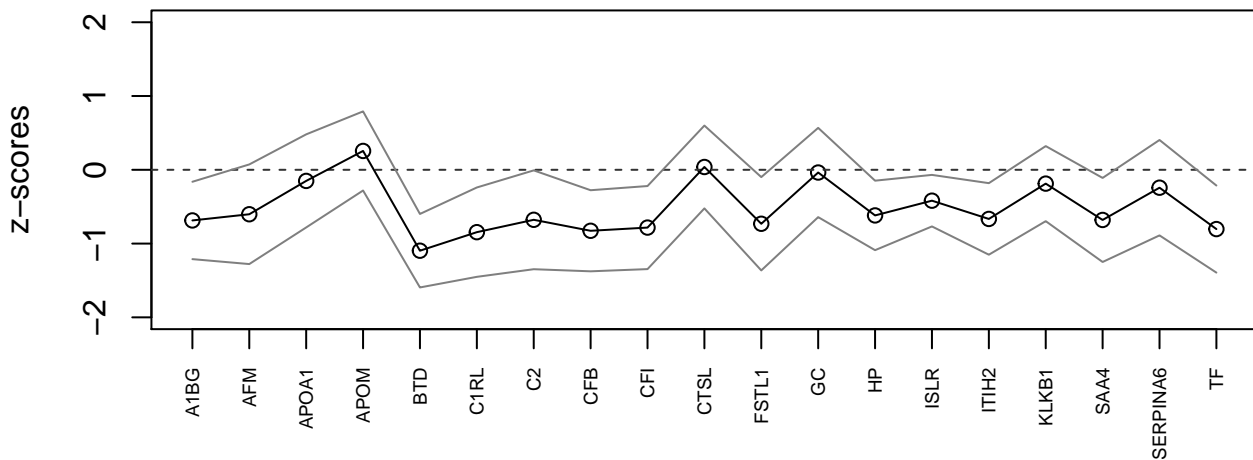

**(b)**

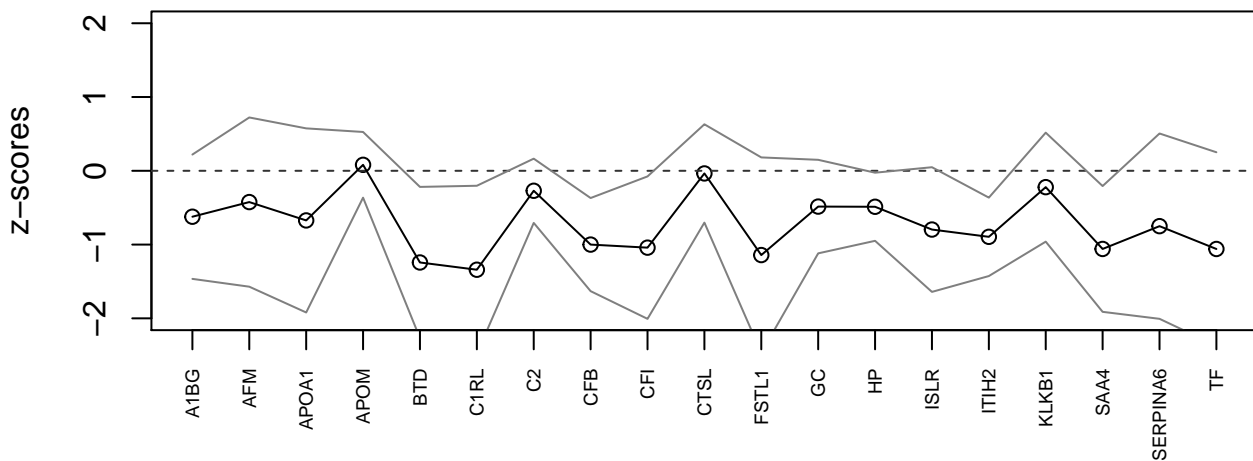

Supplement: Supplementary file 4 — Figure S4. [file 41598_2021_82388_MOESM4_ESM.pdf]

**(a)**

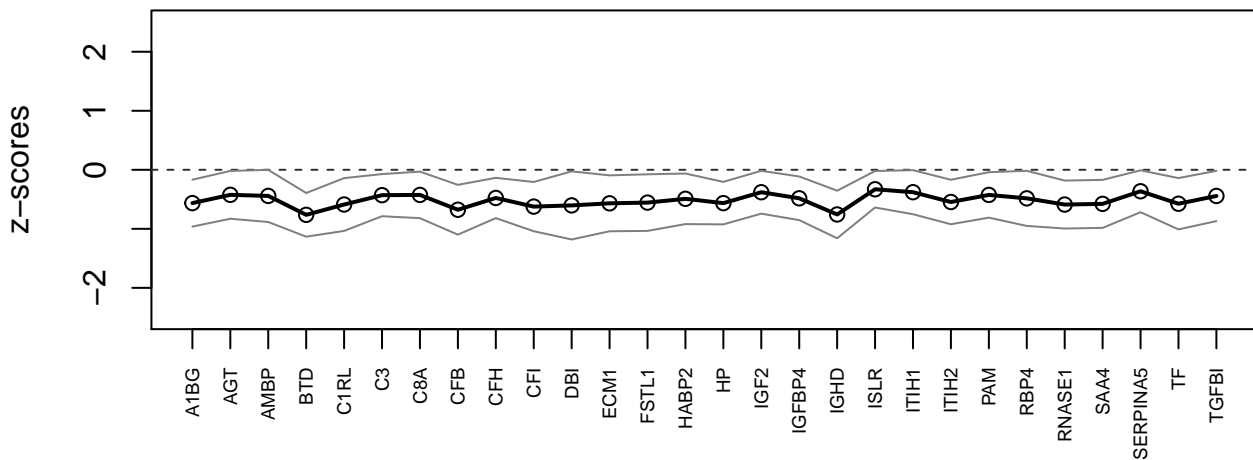

**(b)**

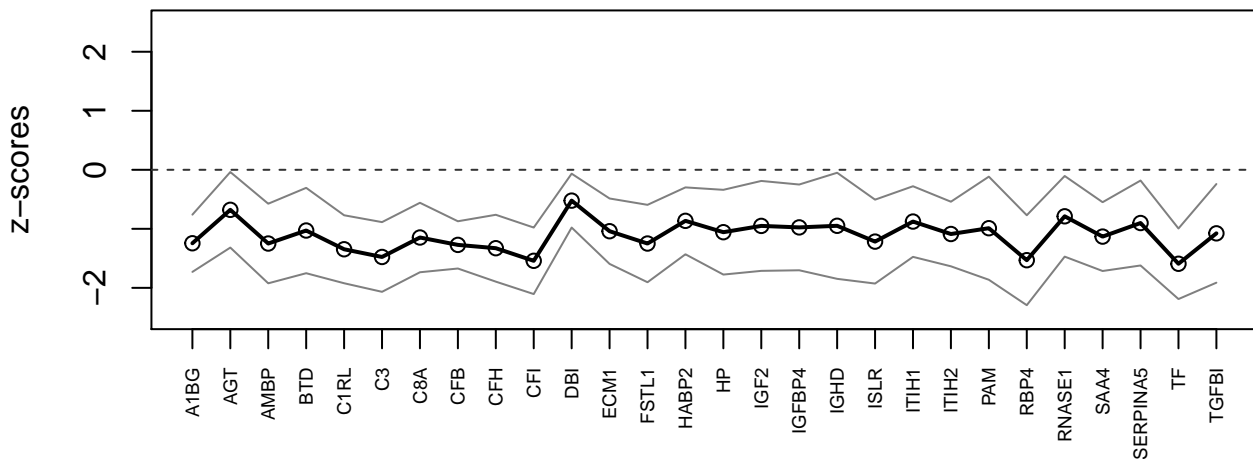

Supplement: Supplementary file 5 — Figure S5. [file 41598_2021_82388_MOESM5_ESM.pdf]

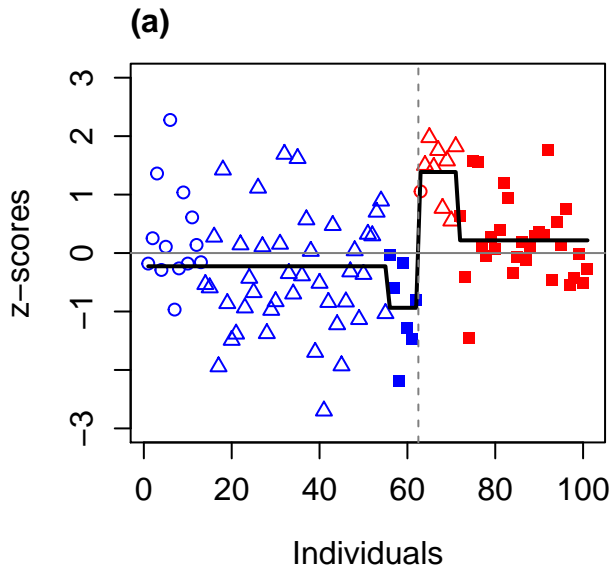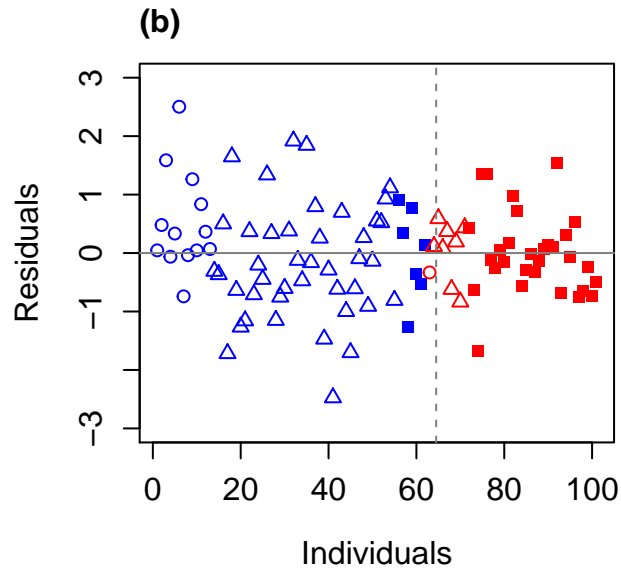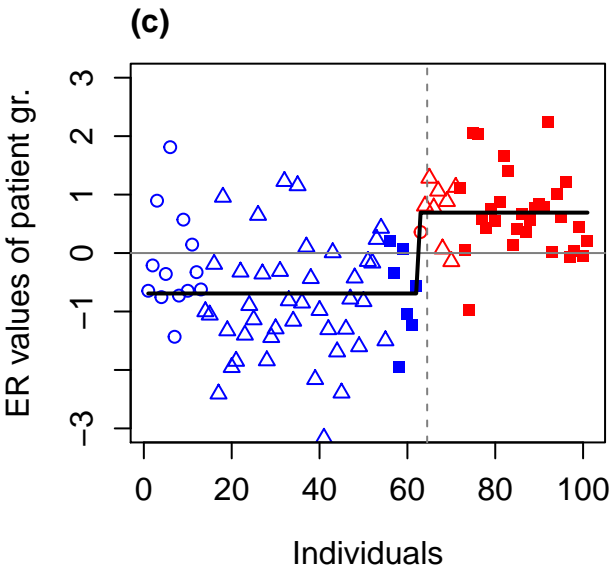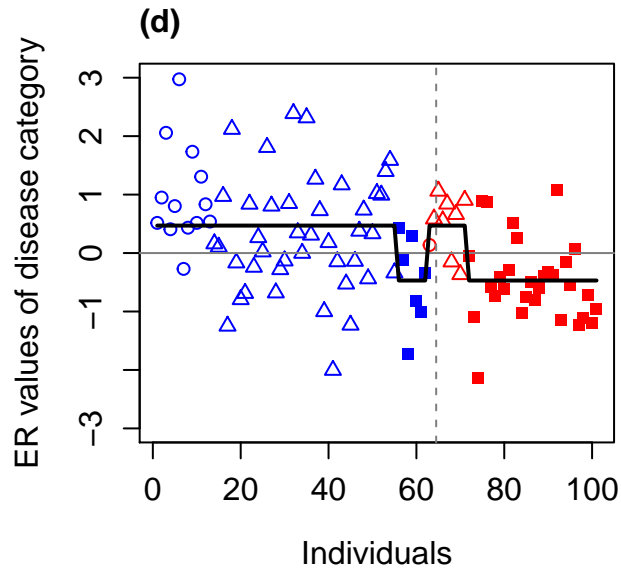

Supplement: Supplementary file 6 — Figure S6. [file 41598_2021_82388_MOESM6_ESM.pdf]

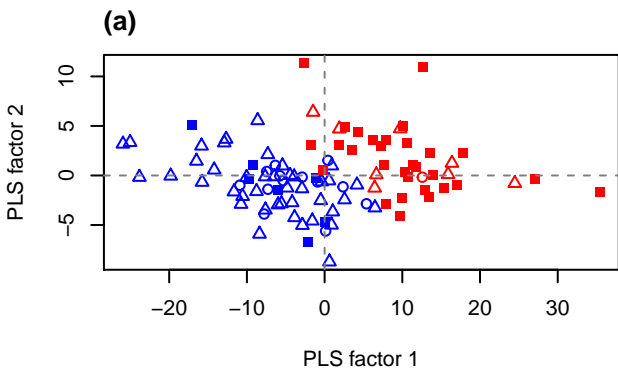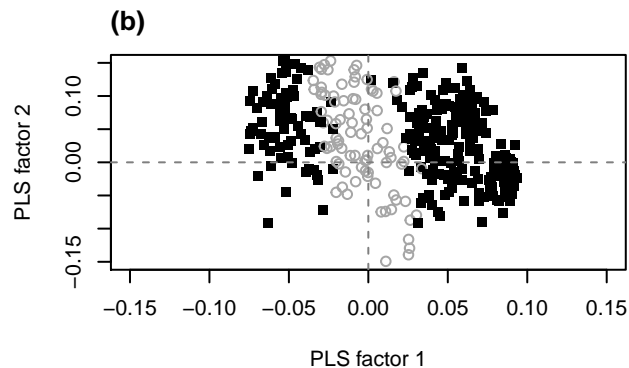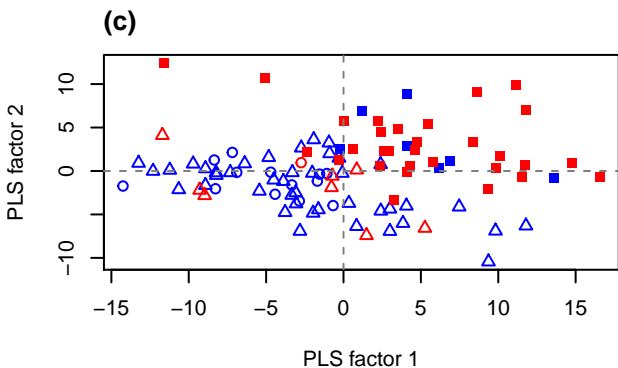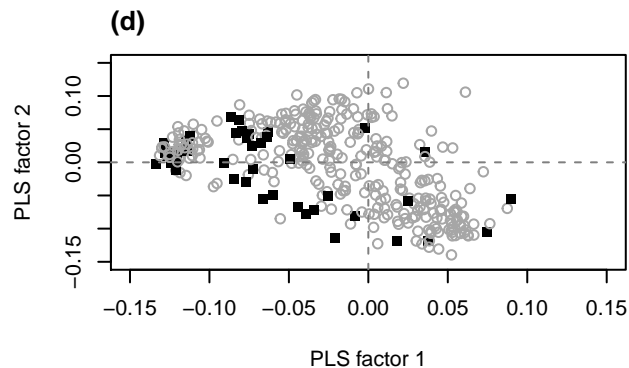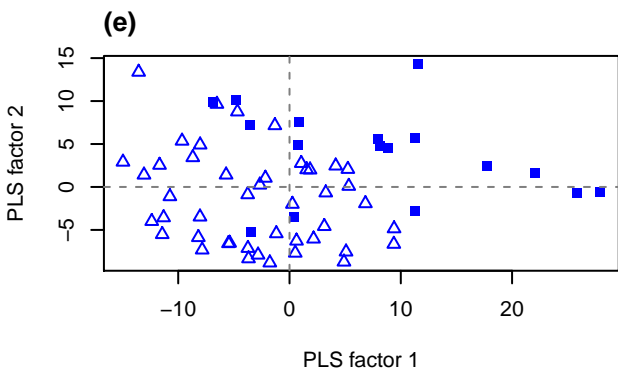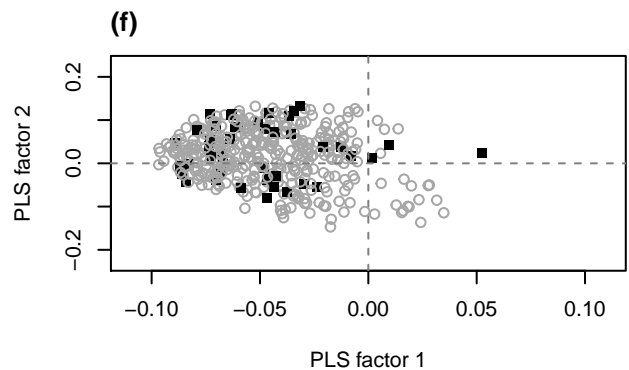

Supplement: Supplementary file 7 — Figure S7. [file 41598_2021_82388_MOESM7_ESM.pdf]

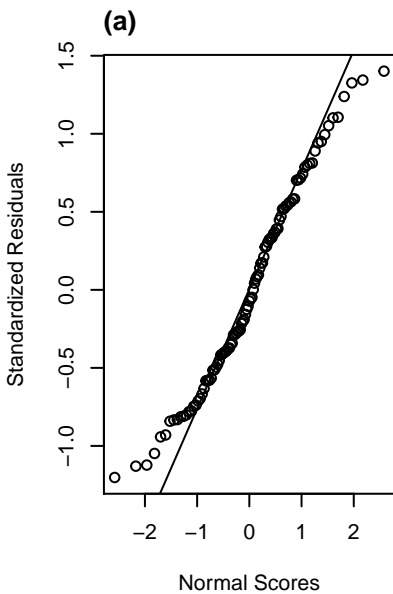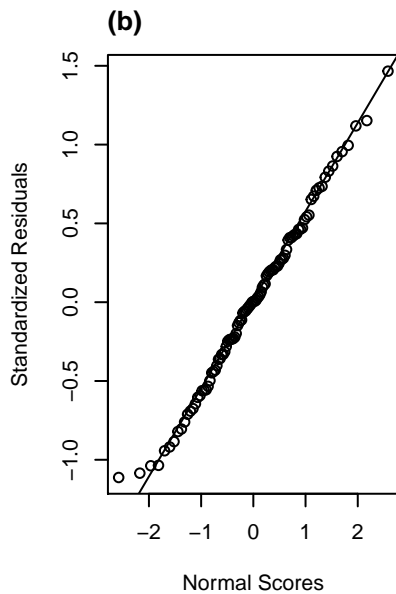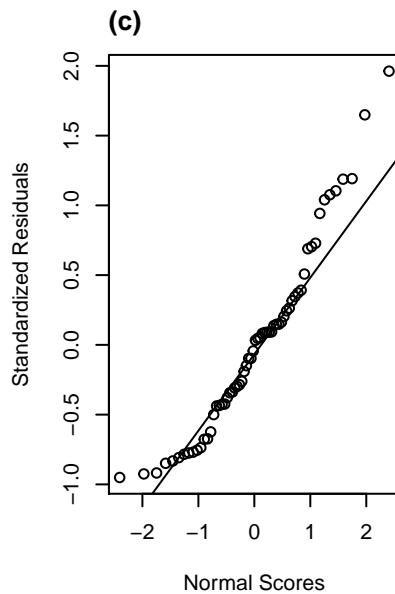

Supplement: Supplementary file 8 — Figure S8. [file 41598_2021_82388_MOESM8_ESM.pdf]

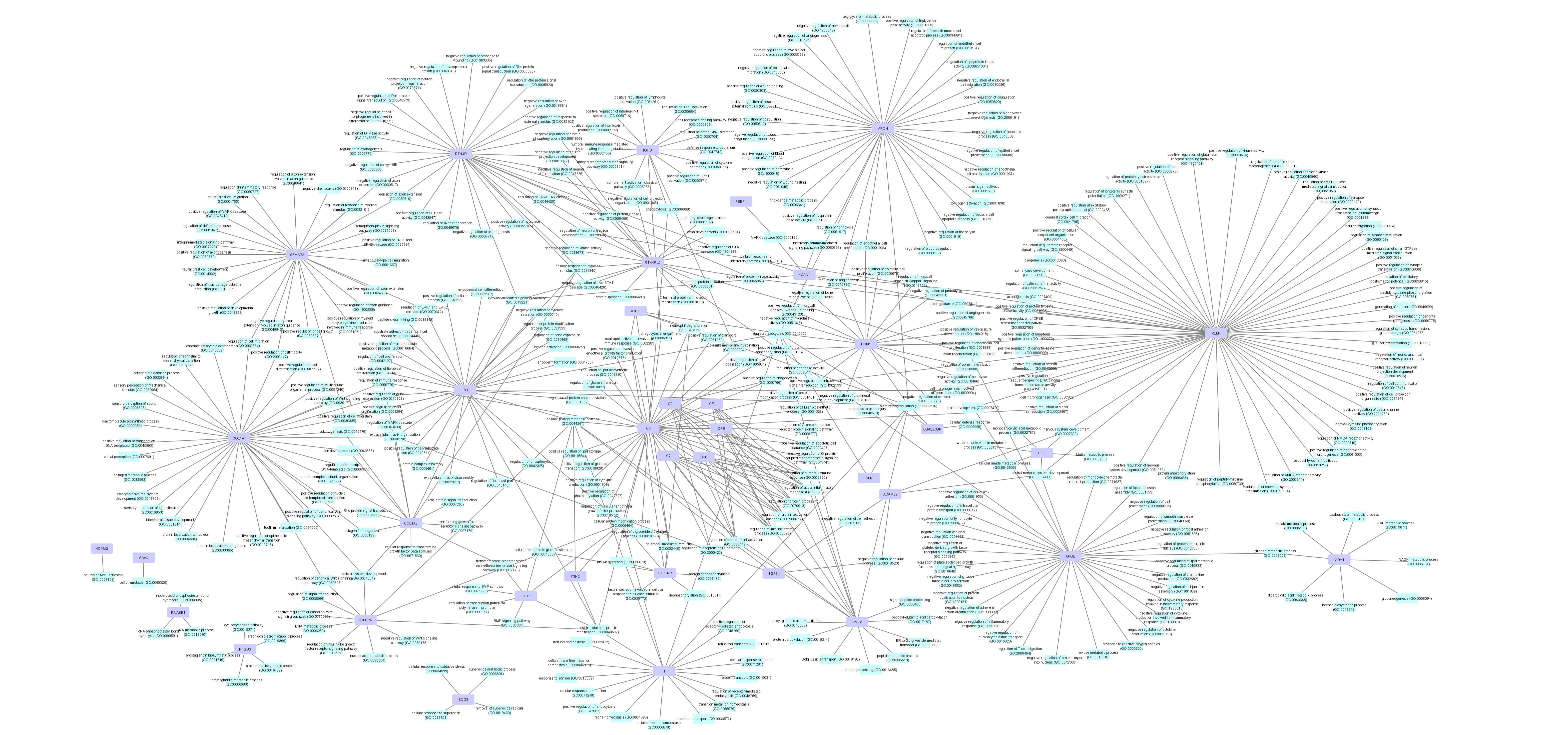

Supplement: Supplementary file 9 — Figure S9. [file 41598_2021_82388_MOESM9_ESM.png]

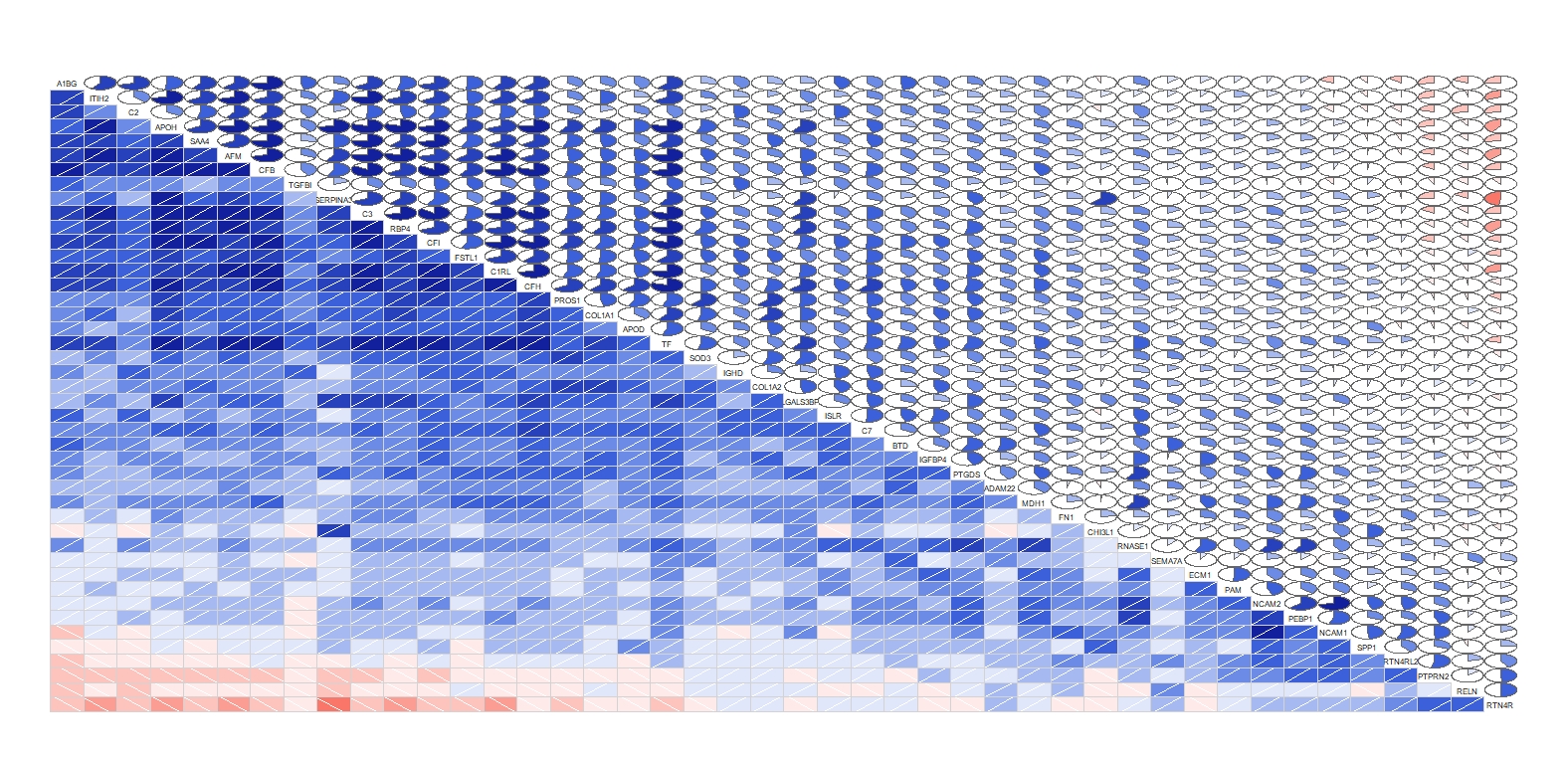

Supplement: Supplementary file 10 — Figure S10. [file 41598_2021_82388_MOESM10_ESM.jpeg]

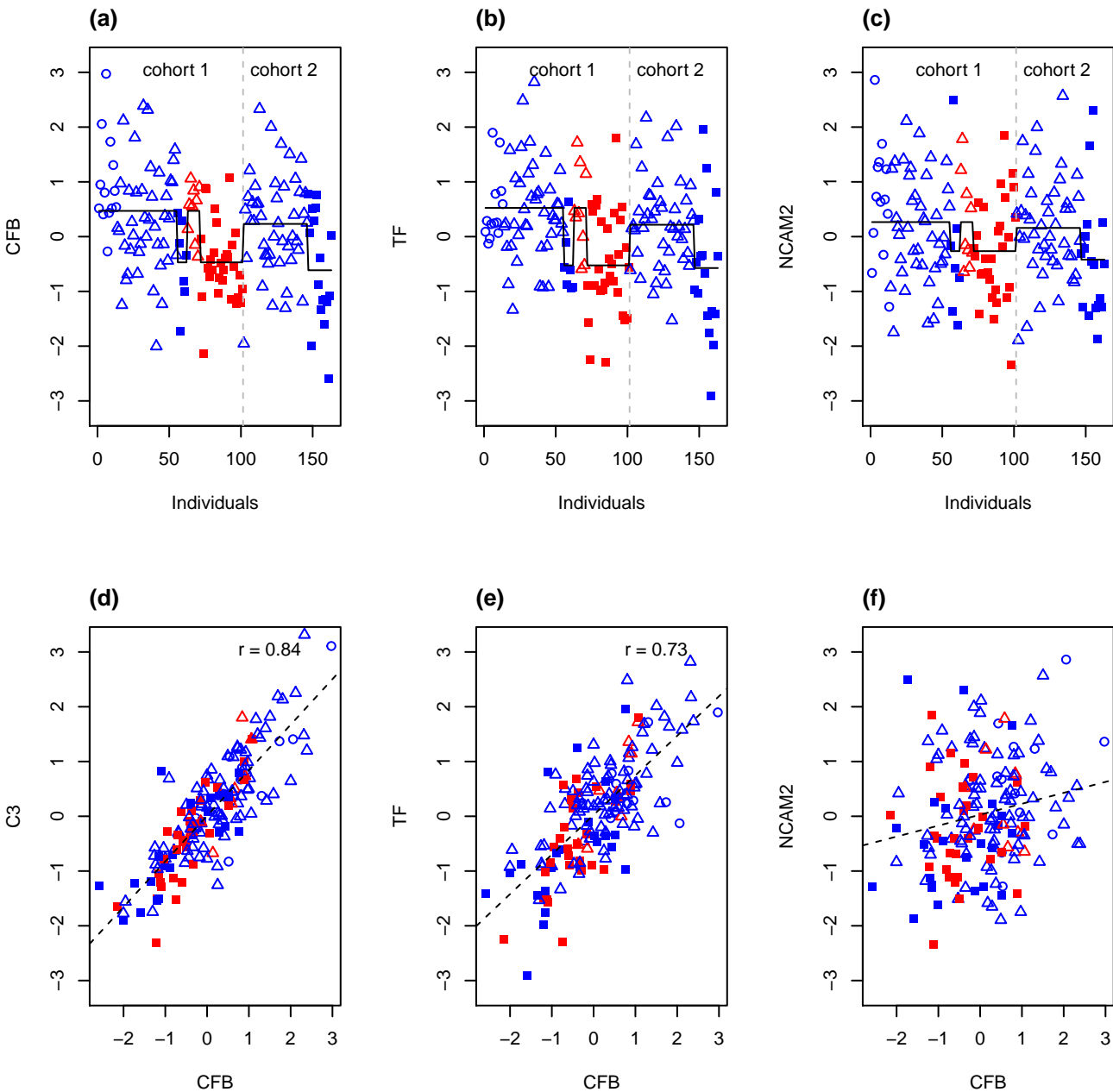

Supplement: Supplementary file 11 — Figure S11. [file 41598_2021_82388_MOESM11_ESM.pdf]

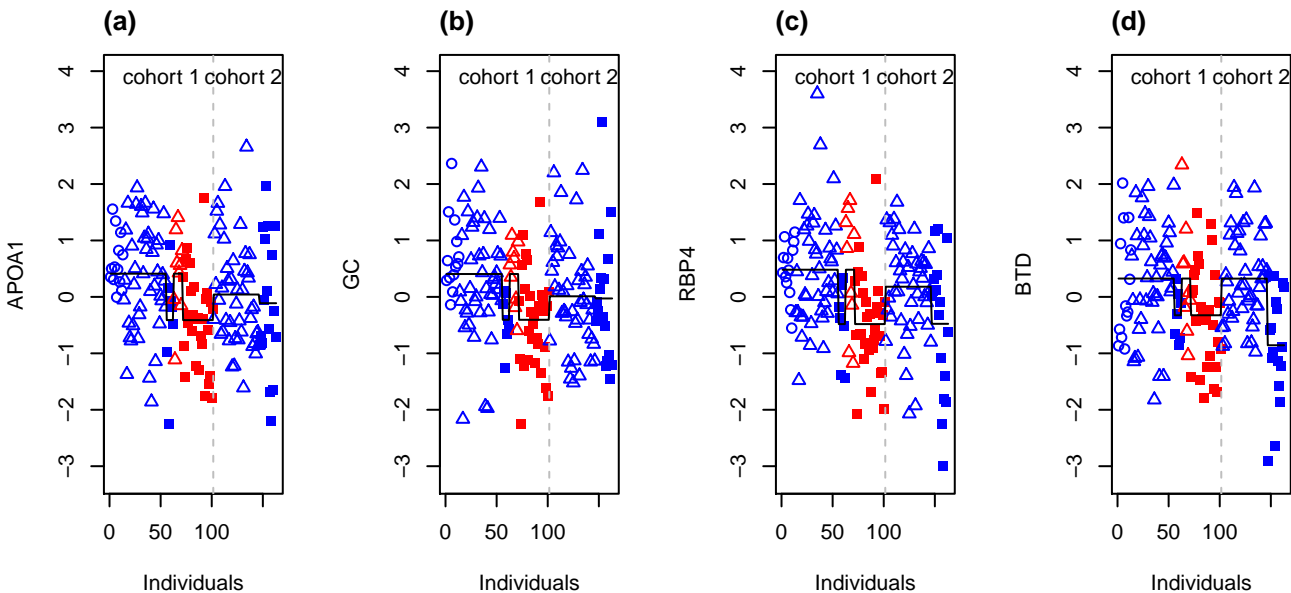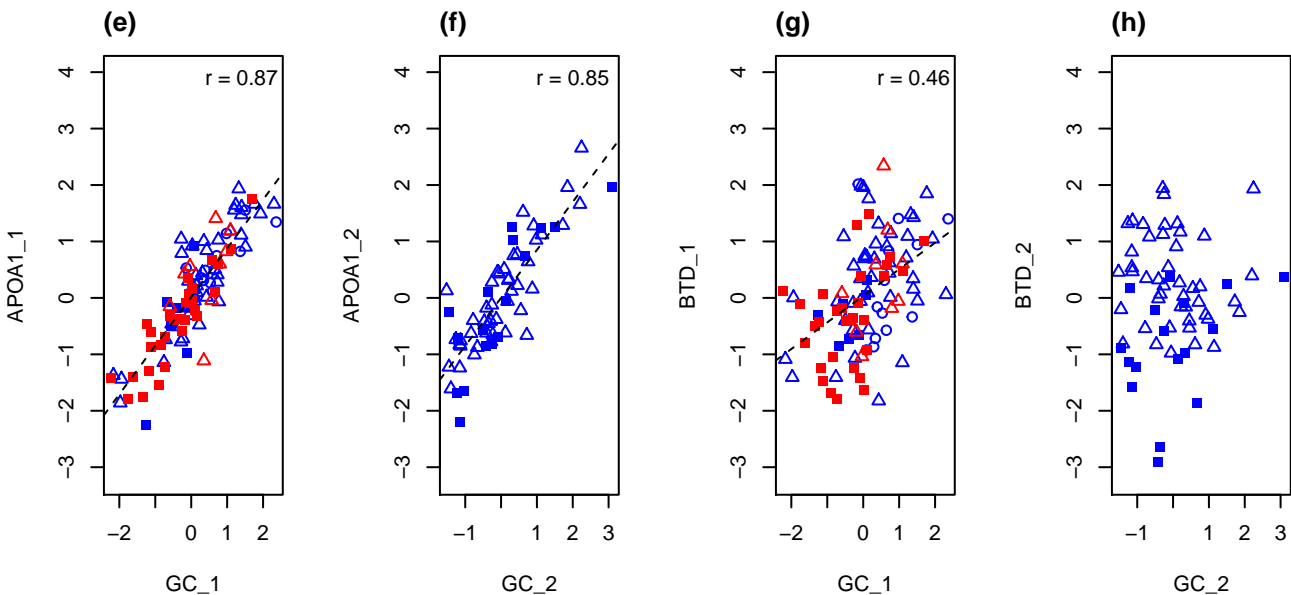

Supplement: Supplementary file 12 — Figure S12. [file 41598_2021_82388_MOESM12_ESM.pdf]

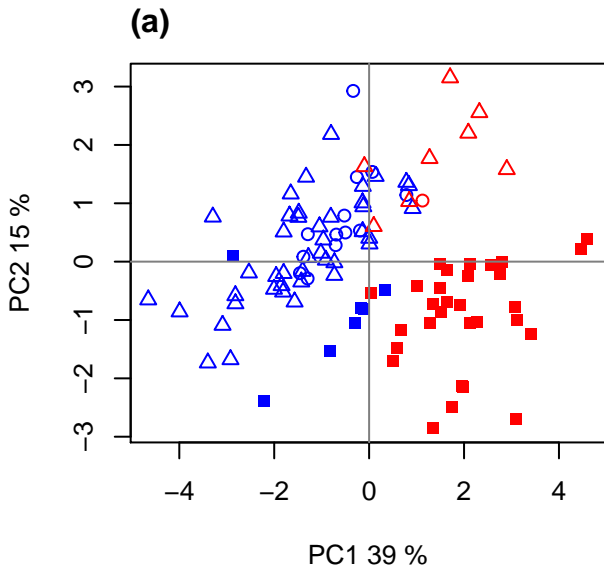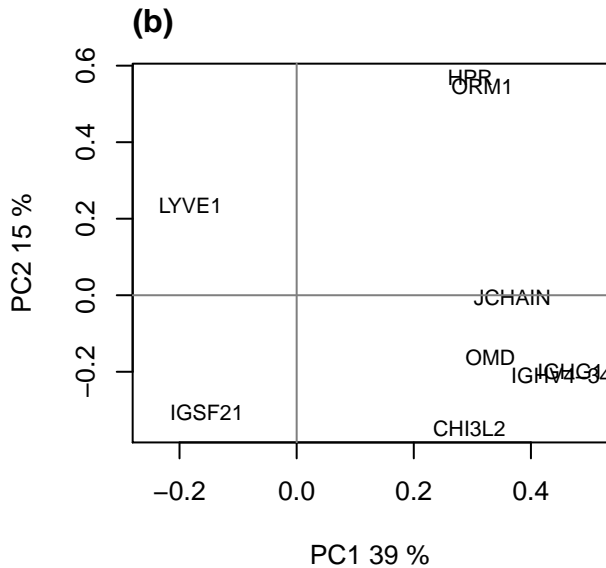

Supplement: Supplementary file 13 — Figure S13. [file 41598_2021_82388_MOESM13_ESM.pdf]

**IGHG1**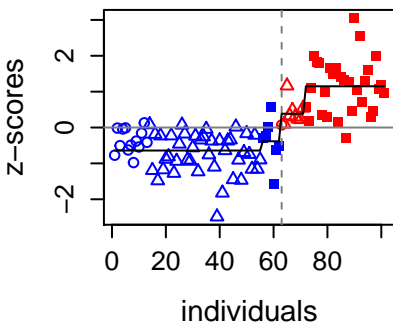**IGHV4-34**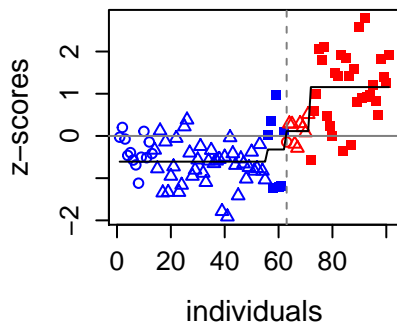**OMD**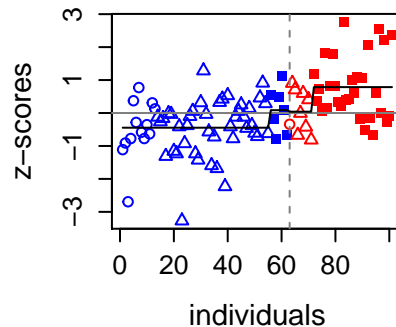**JCHAIN**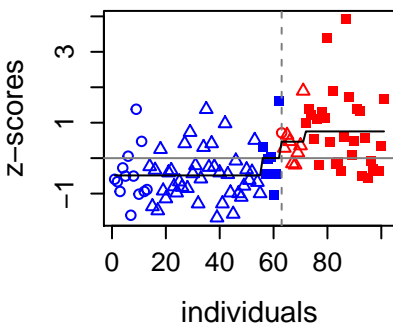**CHI3L2**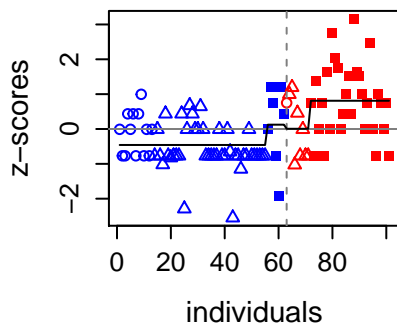**ORM1**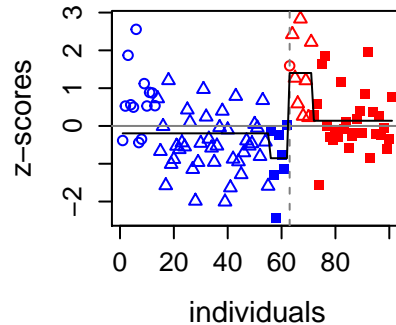**LYVE1**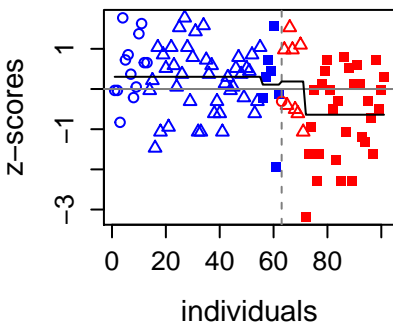**HPR**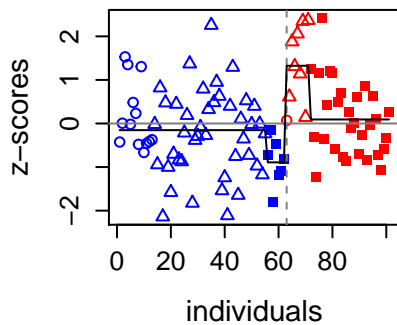**IGSF21**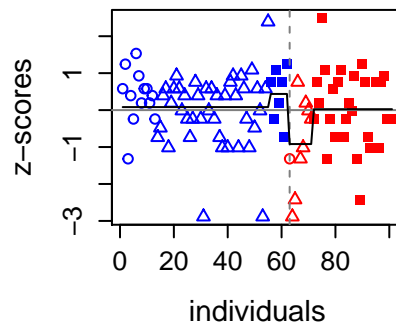

Supplement: Supplementary file 14 — Figure S14. [file 41598_2021_82388_MOESM14_ESM.pdf]
