# Supplementary material for: Cerebrospinal fluid proteome shows disrupted neuronal development in multiple sclerosis
Source: Sci Rep. 2021 Feb 18;11:4087. doi: 10.1038/s41598-021-82388-w (PMC7892850; doi:10.1038/s41598-021-82388-w)
Supplement: Supplementary file 15 — Figure S15. [file 41598_2021_82388_MOESM15_ESM.docx]

**ER Modelling**

Step 1. For each mean centered feature (**y**), a linear model is applied with class variables (**x_1_** and **x_1_**) and their interaction term (**x_12_**) as input, which estimates one regression coefficient (*b_i_*) for each input factor (factor 1, factor 2 and their interaction):

**y** = **x_1_***b_1_* + **x_2_***b_2_* + **x_12_***b_12_* + R (1)

This is repeated for all features in the data (Y) (each the columns in the data table Y) to estimate a vector of regression coefficients (**b_i_**) for each factor:

**Y** = **x_A1_b_1_** + **x_2_b_2_** + **x_12_b_12_** + R (2)

Step 2. Effects (E_i_) are calculated from the estimated regression coefficients (**b_1_ b_2_ b_12_**) by multiplying **x_i_** and **b_i_** to give:

Y = E_1_ + E_2_  + E_12_ + R (3)

The values of Y that predicted from the model ($\hat{Y}$) are sums of the effects:

$\hat{Y}$= E_1_ + E_2_  + E_12_  (4)

Step 3. Data tables are constructed, one for each term in the model, as the sums of the effects of each term plus the residuals of the whole model, and these are called Effect plus Residual (E_i_R) values:

E_1_R = E_1_ + R (5)
E_2_R = E_2_ + R (6)
E_12_R = E_12_ + R (7)

Step 4. Statistical discriminant analysis for each model term can be performed with the ER values as the input and the class variable **x_i_** as the response:

**X_1_** = E_1_R (8)
**x_2_** = E_2_R (9)
**x_12_** = E_12_R (10)

*Matrixes are in capital letters, vectors are in bold small letters, and scalars in cursive.*

*The ER values can be utilised in downstream analysis, such as discriminant analyses (equations 8-10) or other analysis suitable for the data at hand. When appropriate, the degrees of freedom are adjusted for the deflated input factors in the linear models (equation 1-3).*

*When relevant, more main factors and interactions may be included in the linear model.*
