# Supplementary material for: Cerebrospinal fluid proteome shows disrupted neuronal development in multiple sclerosis
Source: Sci Rep. 2021 Feb 18;11:4087. doi: 10.1038/s41598-021-82388-w (PMC7892850; doi:10.1038/s41598-021-82388-w)
Supplement: Supplementary file 16 — Figure S16. [file 41598_2021_82388_MOESM16_ESM.docx]

**ER modelling**

# Factor 1 and factor 2 are two input factors in a linear model.
# For our cohort 1:
 # factor 1 is group affiliation: group A (level -1) and group B (level +1)
 # factor 2 is disease status: controls (level -1) MS (level +1)
 # features are the proteome data mean centred and scaled to unit variance

# Organise the data into an array

my.array <- data.frame(
 factor1=factor1,
 factor2=factor2,
 features=I(features))

# ER modelling

ER.mod <- ER(features ~ factor1*factor2, data = my.array)
ER.values.1 <- as.data.frame(unclass(ER.mod$ER.values$factor1))
ER.values.2 <- as.data.frame(unclass(ER.mod$ER.values$factor2))
ER.values.12 <- as.data.frame(unclass(ER.mod$ER.values$`factor1:factor2`))

# PLS-DA model in ER modelling with feature extraction by Jackknife
my.ncomp <- 2 *# choose the appropriate number of PLS factors for the data at hand*
pls.mod <- pls(ER.mod, 'factor2', ncomp= my.ncomp,
 validation='LOO', jackknife = TRUE, df.used = 1)
scores <- scores(pls.mod) *# scores of the samples*loadings <- loadings(pls.mod) *# loadings of the features*residuals <- pls.mod$residuals[,1, my.ncomp]
p.jkn <- pls.mod$jack[,1,my.ncomp] *# Martens’ Uncertainty Test for feature selection in PLS*
qqnorm(residuals,ylab="Standardized Residuals", xlab="Normal Scores",main='')
qqline(residuals) *# normal probability plot of the residuals*

# Confidence intervals
# Testing the difference between the two class levels one factor within both class levels of the other factor
(illustrated for factor 2 within factor 1 = 1)
conf.factor2.within.factor1.level1 <- with(my.array, confints(features[factor2 == -1 & factor1 == 1,],
 features[factor2 == 1 & factor1 == 1,]))
