# Supplementary material for: Cerebrospinal fluid proteome shows disrupted neuronal development in multiple sclerosis
Source: Sci Rep. 2021 Feb 18;11:4087. doi: 10.1038/s41598-021-82388-w (PMC7892850; doi:10.1038/s41598-021-82388-w)
Supplement: Supplementary file 21 — Figure S17. [file 41598_2021_82388_MOESM21_ESM.docx]

import pandas as pd

import numpy as np

import matplotlib.pyplot as plt

from sklearn.metrics import roc_auc_score

from sklearn.model_selection import cross_val_score

from sklearn.feature_selection import RFECV

from sklearn.linear_model import LogisticRegression

from sklearn.svm import SVC

from sklearn.decomposition import PCA

df = pd.read_excel('https://nofima.no/filearchive/publications/2020/multiple_sclerosis/DM.1.xlsx')

df.drop('Unnamed: 0', axis = 1, inplace = True)

df.head()

target = []

for a,b in zip(df['A'],df['B']):

    if a == 1 and b == 1: # INF MS ---> red quare

        target.append(4)

    elif a == 1 and b == -1: # INF No MS ----> red triangle

        target.append(3)

    elif a == -1 and b == 1: # No INF MS ----> blue square

        target.append(2)

    elif a == -1 and b == -1: # No INF No MS ---> blue Triangle

        target.append(1)

target = pd.Series(target) # used in pca plotting

MS = df['B'] # used in RFECV

df.drop(['A', 'B'], axis = 1, inplace = True)

def protein_finder(y , estim, df): # estim is the model chosen

    x = df.values

    rfecv = RFECV(estimator= estim, step=1, cv=7, scoring='roc_auc', n_jobs = -1)

    rfecv.fit(x, y)

    plt.figure()

    plt.xlabel("Number of features selected")

    plt.ylabel("Cross validation score (AUC)")

    plt.plot(range(1, len(rfecv.grid_scores_) + 1), rfecv.grid_scores_)

    plt.show()

    feature_importance = list(zip(df.columns, rfecv.support_))

    new_features = []

    for key,value in enumerate(feature_importance):

        if(value[1]) == True:

            new_features.append(value[0])

    print('Proteins selected: ',new_features)

    print('total features selected: ',len(new_features))

    print('the model used: ', estim)

    return new_features

def pca_plot(x):

    if x.shape[1] > 1:

        x = x.values

        pca = PCA(n_components=2)

        principalComponents = pca.fit_transform(x)

        principalDf = pd.DataFrame(data = principalComponents,

                                    columns = ['PCA1', 'PCA2'])

        principalDf['group'] = target

        fig = plt.figure(figsize = (10,10))

        ax = fig.add_subplot(1,1,1)

        ax.set_xlabel('First principal component', fontsize = 15)

        ax.set_ylabel('Second principal component', fontsize = 15)

        ax.set_title('2 component PCA', fontsize = 20)

        targets = [1,2,3,4]

        for val in targets:

            if val ==1: # blue triangle

                indicesToKeep = principalDf['group'] == val

                ax.scatter(principalDf.loc[indicesToKeep, 'PCA1'],

                            principalDf.loc[indicesToKeep, 'PCA2'], marker='^',

                            facecolors='none', edgecolors='b')

            if val ==2:

                indicesToKeep = principalDf['group'] == val

                ax.scatter(principalDf.loc[indicesToKeep, 'PCA1'],

                            principalDf.loc[indicesToKeep, 'PCA2'],

                            marker='s', edgecolors='b')

            if val == 3: # red triangle empty

                indicesToKeep = principalDf['group'] == val

                ax.scatter(principalDf.loc[indicesToKeep, 'PCA1'],

                            principalDf.loc[indicesToKeep, 'PCA2'],

                            marker='^', facecolors='none', edgecolors='r')

            if val == 4:

                indicesToKeep = principalDf['group'] == val

                ax.scatter(principalDf.loc[indicesToKeep, 'PCA1'],

                            principalDf.loc[indicesToKeep, 'PCA2'],

                            marker='s', c = 'r')

    else:

        print('x shape is one or less')

models = [LogisticRegression(solver = 'lbfgs', max_iter = 2000, random_state = 1), # Model 1

          LogisticRegression(solver = 'liblinear', random_state = 1), # Model 2

          SVC(kernel = 'linear', random_state = 1), # Model 3

         ]

x_train, x_test, y_train, y_test = train_test_split(df,MS, test_size = 0.1, random_state = 1, stratify = MS)

for model in models:

    proteins = protein_finder(MS,model,df)

    pca_plot(x = df[proteins])

    model.fit(x_train[proteins],y_train)

    print('model test performance: ', roc_auc_score(model.predict(

x_test[proteins]),y_test))
