# Supplementary material for: Cerebrospinal fluid proteome shows disrupted neuronal development in multiple sclerosis
Source: Sci Rep. 2021 Feb 18;11:4087. doi: 10.1038/s41598-021-82388-w (PMC7892850; doi:10.1038/s41598-021-82388-w)
Supplement: Supplementary file 22 — Supplementary Legends. [file 41598_2021_82388_MOESM22_ESM.pdf]

Supplementary legends

Fig. S1. Cohort 1. Confidence intervals of proteins significant for MS versus controls within both groups displayed as (a) the differences of MS versus controls within group A, where the differences are in solid black lines marked by a circle for each protein along the x-axis, and the grey lines are 95% confidence interval boarders, (b) the differences of MS versus controls within group B, (c) the differences of MS versus controls within group A where the controls are only those without neurological disorders. The protein names are provided in Supplementary Table S2.

Fig. S2. Cohort 1. A graph of proteins significant for group affiliation by confidence intervals (95%) for MS patients and controls (in the horizontal direction, elevated for group B towards right and elevated for group A toward left), and proteins significant for MS versus controls of both groups (in the vertical direction, downwards for proteins lower in abundance for MS versus controls). Gene symbols label the nodes (purple) with protein names provided in Supplementary Table S2. Additional annotations for these proteins are displayed for molecular functions (pink nodes), biological processes (cyan nodes), and KEGG metabolic pathways [17-19] (green nodes).

Fig. S3. Cohort 2. The abundance of Ig kappa chain C region (IGKC) along the y-axis, expressed as z-scores after mean cantering and standardization, was elevated for CIS patients only in group 2. (a) z-scores of IGKC for group A (blue) displaying controls (open triangles) and CIS patients (closed squares) and (b) z-scores of IGKC for group B (red) comprised only of CIS patients (closed squares). The individuals within each group are listed along the x-axes.

Fig. S4. Cohort 2. Confidence intervals (95%) of proteins identified as significant for MS versus controls in cohort 1, validated for cohort 2. (a) The significant proteins displayed as confidence intervals for the differences between CIS patients versus controls in group A of cohort 2. The mean differences are in solid black lines marked by a circle for each protein along the x-axis, and the grey lines are 95% confidence interval boarders. (b) Corresponding confidence interval plots as in panel a including only the CIS patients in group A who converted to MS during the follow-up time of 10 years. The protein names are provided in Supplementary Table S2.

Fig. S5. Cohorts 1 and 2. (a) Confidence intervals (95%) of the differences between MS/CIS patients and controls for all individuals in group A after merging the data for the two cohorts without any adjustment within or between the cohorts. (b) Confidence intervals (95%) of the differences between MS/CIS patients and controls for all individuals in group B in data combined for the two cohorts. The protein names are provided in Supplementary Table S2.

Fig. S6. Cohort 1. ER modelling, illustrated for CFB, isolates the effects of group affiliation and the effects of MS status. In each figure, along the x-axis are group A (blue; to the left of the hatched line) and group B (red; to the right). Within each group from the left are controls without other neurological disorders (circles), controls with neurological disorders (triangles), and MS patients (squares). (a) The data (as points) expressed as z-scores obtained by subtracting means and dividing by standard deviation, and the predicted values (as horizontal line), (b) the residuals, (c) ER values of group affiliation (as points) and the effects of group (as horizontal lines), (d) ER values of disease categories (as points) and the effects of disease categories (as horizontal lines).

Fig. S7. Cohorts 1 and 2. Supervised multivariate analysis by PLS discriminant analysis (PLS-DA). (a, b) Cohort 1. PLS-DA of ER values that isolate the effects of group affiliation using category indicator variable of group as response displayed by (a) scores of the participants for PLS factor 2 versus 1, (b) the corresponding loadings of the proteins for PLS factor 2 versus 1. This model resulted in 89% correct classification of group affiliation by the first PLS factor as validated by full cross validation. (c, d) Cohort 1. PLS-DA of ER values that isolate the effects of MS status using as response indicator variable for MS and controls, displayed by (c) scores of individuals and (d) the corresponding loadings of the proteins. This model correctly classified 91% of the individuals as MS or controls in a model with four PLS factors (85% in a model with two first PLS factors). (e, f) Cohort 2, PLS-DA of CIS patients versus controls displayed as (e) scores of individuals and (f) the corresponding loadings of the proteins. This model with two PLS factors correctly classified 81% of the individuals as CIS or controls. The score plots (a, c, e) display group A (blue) and group B (red) for controls without other neurological disorders (circles), controls with neurological disorders (triangles), and MS patients (filled squares). In the loading plots (b), the proteins selected by Martens' uncertainty test [23] in PLS-DA are marked in filled black squares; others are in open grey circles, and in the loading plots (d, f) the proteins selected in both cohorts with consistent regression coefficient by the two first PLS factors are marked. Protein names are listed in Supplementary Table S4.

Fig. S8. Cohort 1 and 2. Normal probability plots of the PLS-DA models presented in Supplementary Fig. S7 (a) PLS-DA of ER values of the group affiliation in cohort 1 with category indicator variable of group as response, (b) PLS-DA of ER values of the disease category in cohort 1 applied to discriminate MS patients versus controls with category indicator variable of MS status as response (c) PLS-DA of CIS patients versus controls in cohort 2 with category indicator variable of CIS status as response, applied directly on the data as there is only one group.

Fig. S9. Cohort 1 and 2. A graph of 44 proteins selected by multivariate analysis of each cohort (1 and 2) labelled with gene symbols (purple nodes), with protein names provided in Supplementary Table S4, and the biological processes displayed for each protein (cyan nodes).

Fig. S10. Correlated pattern of selected proteins as analysed across cohorts 1 and 2 where ER values of MS status from cohort 1 is merged with data of group A from cohort 2 resulting in a large data table of 163 individuals. The plot displays simple pairwise correlations between of proteins across all 163 individuals selected to discriminate MS/CIS versus controls within both cohorts. The colour and the filled portion of the pie indicates the magnitude of the correlation. The protein names are given in Table 2 and Supplementary Table S4.

Fig. S11. Plots of selected proteins across the two cohorts (1 and 2), where ER values of MS status from cohort 1 is merged with data of group A from cohort 2 resulting in a large data table of 163 individuals. One-dimensional plots of (a) CFB, (b) TF, (c) NCAM2, and two-dimensional plots of (d) C3 versus CFB, (e) TF versus CFB, and (f) NCAM2 versus CFB. Codes are group A (blue) and group B (red) for controls without other neurological disorders (circles), controls with neurological disorders (triangles), and MS patients (filled squares). In panel (a), (b), (c) individuals from cohort 1 are to the left of the hatched line, and those in cohort 2 are to the right.

Fig. S12. Plots of selected proteins as analysed across the two cohorts (1 and 2), where ER values of MS status from cohort 1 is merged with data of group A from cohort 2 resulting in a large data table of 163 individuals. One-dimensional plots of (a) APOA1, (b) GC, (c) RBP4, (d) BTD and two-dimensional plots of

(e) APOA1 versus GC within cohort 1, (f) APOA1 versus GC within cohort 2, (g) BTD versus GC within cohort 1, (h) BTD versus GC within cohort 2. Codes are group A (blue) and group B (red) for controls without other neurological disorders (circles), controls with neurological disorders (triangles), and MS patients (filled squares). In panel (a), (b), (c), and (d) individuals from cohort 1 are to the left of the hatched line, and those in cohort 2 are to the right.

Fig. S13. Cohort 1. Principal Component Analysis (PCA) of proteins selected to separate MS patients versus controls is influenced by the group affiliation, although the model was only trained for the disease categories (MS versus controls). (a) Score plot of PC2 versus PC1 displaying group A (blue) and group B (red) for controls without other neurological disorders (circles), controls with neurological disorders (triangles), and MS patients (filled squares). (b) The corresponding loadings of PC2 versus PC1. IGHG1 overlap with IGHV4-34, and ORM1 overlap with HPR. The corresponding protein names are given in Supplementary Table 2.

Fig. S14. Cohort 1. One-dimensional plots of the proteins included in the PCA plot in Supplementary Fig. S13. The y-axes are the protein levels expressed as z-scores, obtained by subtracting means and dividing by standard deviation, and the x-axes are the participants in the cohort. Along the x-axis are group A (blue; to the left of the hatched line) and group B (red; to the right). Within each group from the left are controls without other neurological disorders (circles), controls with neurological disorders (triangles), and MS patients (filled squares). The horizontal solid lines indicate mean values within each of the four combinations of group affiliation and disease category.

Fig. S15. ER modelling of data with two factors and their interaction term.

Fig. S16. R scripts to run ER modelling on one dataset using the program 'ER'.

Figure S17. Python code used for the multivariate analysis of MS status ignoring group affiliation.
